# Techno-economic analysis and life cycle assessment of a biorefinery utilizing reductive catalytic fractionation†

**DOI:** 10.1039/d1ee01642c

**Published:** 2021-07-08

**Authors:** Andrew W. Bartling, Michael L. Stone, Rebecca J. Hanes, Arpit Bhatt, Yimin Zhang, Mary J. Biddy, Ryan Davis, Jacob S. Kruger, Nicholas E. Thornburg, Jeremy S. Luterbacher, Roberto Rinaldi, Joseph S. M. Samec, Bert F. Sels, Yuriy Román-Leshkov, Gregg T. Beckham

**Affiliations:** Catalytic Carbon Transformation and Scale-Up Center, National Renewable Energy Laboratory Golden CO 80401 USA gregg.beckham@nrel.gov; Center for Bioenergy Innovation Oak Ridge TN 37830 USA; Department of Chemical Engineering, Massachusetts Institute of Technology Cambridge MA 02139 USA yroman@mit.edu; Strategic Energy Analysis Center, National Renewable Energy Laboratory Golden CO 80401 USA; Renewable Resources and Enabling Sciences Center, National Renewable Energy Laboratory Golden CO 80401 USA; Laboratory of Sustainable and Catalytic Processing, Institute of Chemical Sciences and Engineering École Polytechnique Fédérale de Lausanne (EPFL) CH-1015 Lausanne Switzerland; Department of Chemical Engineering, Imperial College London South Kensington Campus London SW7 2AZ UK; Department of Organic Chemistry, Stockholm University SE-106 91 Stockholm Sweden; Center for Sustainable Catalysis and Engineering KU Leuven, Celestijnenlaan 200F 3001 Leuven Belgium

## Abstract

Reductive catalytic fractionation (RCF) is a promising approach to fractionate lignocellulose and convert lignin to a narrow product slate. To guide research towards commercialization, cost and sustainability must be considered. Here we report a techno-economic analysis (TEA), life cycle assessment (LCA), and air emission analysis of the RCF process, wherein biomass carbohydrates are converted to ethanol and the RCF oil is the lignin-derived product. The base-case process, using a feedstock supply of 2000 dry metric tons per day, methanol as a solvent, and H_2_ gas as a hydrogen source, predicts a minimum selling price (MSP) of crude RCF oil of $1.13 per kg when ethanol is sold at $2.50 per gallon of gasoline-equivalent ($0.66 per liter of gasoline-equivalent). We estimate that the RCF process accounts for 57% of biorefinery installed capital costs, 77% of positive life cycle global warming potential (GWP) (excluding carbon uptake), and 43% of positive cumulative energy demand (CED). Of $563.7 MM total installed capital costs, the RCF area accounts for $323.5 MM, driven by high-pressure reactors. Solvent recycle and water removal *via* distillation incur a process heat demand equivalent to 73% of the biomass energy content, and accounts for 35% of total operating costs. In contrast, H_2_ cost and catalyst recycle are relatively minor contributors to operating costs and environmental impacts. In the carbohydrate-rich pulps, polysaccharide retention is predicted not to substantially affect the RCF oil MSP. Analysis of cases using different solvents and hemicellulose as an *in situ* hydrogen donor reveals that reducing reactor pressure and the use of low vapor pressure solvents could reduce both capital costs and environmental impacts. Processes that reduce the energy demand for solvent separation also improve GWP, CED, and air emissions. Additionally, despite requiring natural gas imports, converting lignin as a biorefinery co-product could significantly reduce non-greenhouse gas air emissions compared to burning lignin. Overall, this study suggests that research should prioritize ways to lower RCF operating pressure to reduce capital expenses associated with high-pressure reactors, minimize solvent loading to reduce reactor size and energy required for solvent recovery, implement condensed-phase separations for solvent recovery, and utilize the entirety of RCF oil to maximize value-added product revenues.

## Abstract 2

Analysis of a promising lignin-first biorefining technique, reductive catalytic fractionation, provides useful metrics for cost and sustainability to guide researchers toward critical areas for improvement.

## Graphical abstract

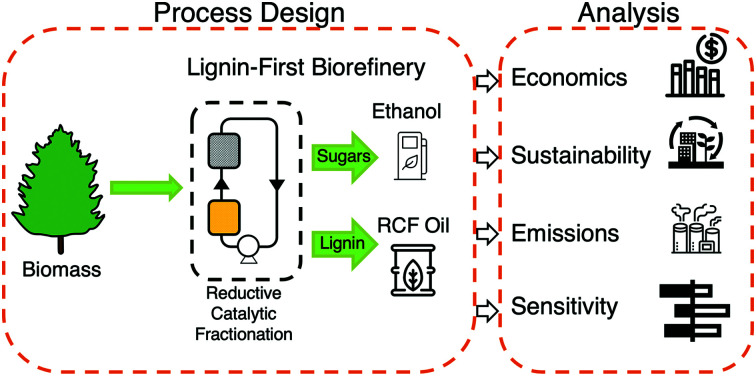

**Broader context**

To enable a viable bioeconomy based on lignocellulosic biomass, all major biomass fractions must be converted to value-added products. Polysaccharides have long been studied and conversion routes for carbohydrates to fuels, chemicals, and materials are under intense development. Conversely, the aromatic polymer, lignin, which comprises a major fraction of carbon in plants, remains a challenge to convert to valuable products, despite a century of research. The concept of reductive catalytic fractionation (RCF) has recently emerged as a potential biorefining strategy to process lignin in its native form, which is the form most conducive to valorization. Economic and sustainability analyses of RCF processes provide quantitative estimates of the major cost and environmental sustainability drivers in an RCF process to inform future research. This work identifies key parameters that exhibit substantial influence on the lignin product selling price and proposes potential solutions to improve the favorability of this promising approach in the context of an integrated biorefinery.

## Introduction

Lignocellulosic biomass is an abundant and environmentally sustainable carbon source capable of expanding global opportunities to produce renewable fuels and chemicals. Towards that goal, a historical focus of lignocellulosic biorefineries has typically aimed to produce ethanol from sugars liberated from plant polysaccharides. However, techno-economic analysis (TEA – see ESI† for a summary of abbreviations used in this study) and life cycle assessment (LCA) of these biorefineries has shown that, barring external incentives, the utilization of lignin (15–30 wt% of biomass composition) as a value-added co-product is essential for commercial viability.^1–6^ However, traditional biomass pretreatment strategies that primarily aim to achieve high yields of fermentable sugars commonly render lignin wastes of condensed, recalcitrant structures that are combusted to produce low-value process heat.^7^ To circumvent the deleterious effects of conventional pretreatment on lignin, the last decade has seen a boom in research on “lignin-first” techniques that can be broadly defined as active stabilization approaches that liberate lignin from the plant cell wall and prevent condensation reactions through either catalysis or protection-group chemistry.^8^

A particularly promising lignin-first technique is reductive catalytic fractionation (RCF), which uses a polar, protic solvent to extract lignin fragments from whole biomass, which are depolymerized and stabilized with a heterogeneous catalyst and hydrogen (or hydrogen donor), generating a lignin product mixture referred to as RCF oil.^9,10^ Research efforts on this technique have demonstrated several unique process configurations and examined the impacts of various reaction parameters. Studies have implemented many catalyst formulations (*e.g.*, Ru,^11^ Pd,^12^ Ni,^13^ Zn–Pd,^14^ Rh,^15^ CuPMO,^16^), solvent compositions (*e.g.* a variety of pure solvents,^17^ mixtures of alcohol and water,^18^ addition of acids or bases,^19^), reducing equivalents (*e.g.* from the solvent^13,20^ or extracted hemicellulose^21^) and a number of different biomass feedstocks.^22–27^ Many of these processes have achieved theoretical maximum yields of monomers from lignin, with tradeoffs such as required reagents, operating pressure, polysaccharide recovery, residence time, and catalyst composition or loading.

Additionally, multiple RCF process configurations are possible.^8^ The most common are batch processes where catalyst and biomass are directly combined in a slurry with the solvent, but RCF has also been demonstrated in a batch process with the catalyst separated in a basket,^28^ flow-through processes where separated beds of biomass and catalyst are aligned in series,^29,30^ or entirely separate extraction and depolymerization processes in which the lignin is stabilized during the extraction step, isolated, and then depolymerized in a second reaction step.^31^ While commonly used batch processes provide effective catalyst contact and the possibility of lower solvent loadings, they are disadvantaged by the difficulty of catalyst separation from the residual pulp and the challenging scale-up of agitated tanks at high operating pressures (*e.g.* 60–100 bar with common conditions of methanol and H_2_ gas at 200–250 °C).^32^ Flow-through configurations are advantaged in progressing towards a continuous mode of operation, removing the need for a catalyst separation from residual pulp, and simplifying reactor internals lacking agitators across a high-pressure envelope. However, solvent requirements are significantly higher than batch operation, presenting a scale-up challenge associated with solvent recovery and equipment sizing.

Compared with conventionally extracted lignin, RCF oil is an attractive lignin-derived feedstock for downstream upgrading due to its high content of monomers, oligomers with low molecular weight and low polydispersity,^33^ non-corrosiveness, high carbon yield, solubility in common solvents, and stability.^34^ Accordingly, several pathways have been proposed for upgrading the RCF lignin oil into commercial products such as fuels,^35,36^ polymers,^37^ adhesives,^38^ commodity chemicals such as phenol and propylene,^39–41^ printer ink,^41^ surfactants,^42,43^ aromatic amines that are building blocks for pharmaceutical and polymer applications,^16^ and various other proposed platform chemicals.^44^ Potential products for RCF oil will need to be considered in the context of the potential product selling price and market volume. Based on the work reported to date, target products have been envisaged from different fractions of the RCF oil (*i.e.*, use of individual monomers, all monomers, or all monomers, dimers, and oligomers). Potential final products from RCF also will likely require different separations and reaction steps to meet requisite specifications. Fortunately, the range of value and volume in potential RCF product opportunities provides flexibility in biorefinery design to meet different economic and sustainability goals.

As RCF research matures, the potential for commercialization of lignin-first biorefining is gaining traction. Recent reports from Liao *et al.* and Tschulkow *et al.* combined several upstream and downstream process steps, including batch RCF and the production of phenol, propylene, and printer ink from lignin products to demonstrate a promising biorefinery concept with the potential for economic feasibility.^41,45^ Beyond this particular study, and in the context of the broad range of potential strategies, it is crucial to examine RCF-based lignin-first biorefining processes in more detail to evaluate process options. To that end, TEA is a powerful tool in guiding early-stage research and process development, providing insights into economic feasibility, scale-up concerns, points of uncertainty, and key impactful parameters for informing research foci. By linking process models to cost drivers, TEA can inform how process changes directly impact the economics and, through sensitivity analyses, provide guidance on which parameters are crucial to optimizing a system and reducing risk in deploying technology. LCA and biorefinery air emissions analysis similarly link process modeling data as well as broader supply chain sourcing of biorefinery material and energy inputs to environmental impacts. Performing LCA concurrently with process simulation allows for the identification of inputs, process areas, and operating parameters that impact sustainability metrics such as global warming potential (GWP) and cumulative energy demand (CED), which can also be targeted for research and process improvements. Utilizing TEA and LCA is critical for biorefinery design, where profitability and environmental sustainability are essential to design constraints.

In this study, we developed a base-case biorefinery model that employs RCF processing with methanol as a solvent and externally-purchased hydrogen, isolating solubilized lignin oil for sale to a subsequent product upgrading operation, coupled with downstream deconstruction and conversion of carbohydrates to ethanol.^46^ A sensitivity analysis evaluating process modifications was then used to identify key cost and environmental impact drivers. Guided by the base-case sensitivity, we explored other RCF configurations from the literature, specifically focusing on the impact of solvent composition and a hydrogen source. Comparing the methanol solvent base-case with ethanol, hydrogen-free, and ethylene glycol cases, this analysis highlights the importance of reagent composition and reaction conditions on capital expenses for high-pressure RCF reactors, as well as associated heat duties required for separations and solvent recycle (the latter of which is responsible for a large share of both environmental impacts and operating costs). Notably, some metrics that have garnered significant focus in the literature, such as catalyst cost, hydrogen consumption, and carbohydrate retention, showed minimal impact on the RCF oil selling price over the range of values considered here. The implications of this work motivate research on minimizing solvent loading, using low vapor pressure solvents, and developing condensed-phase separations. Our models show that these areas can maximize improvements to both TEA and LCA metrics and increase commercial viability.

## Results

### Building a base-case model

**Fig. 1:**
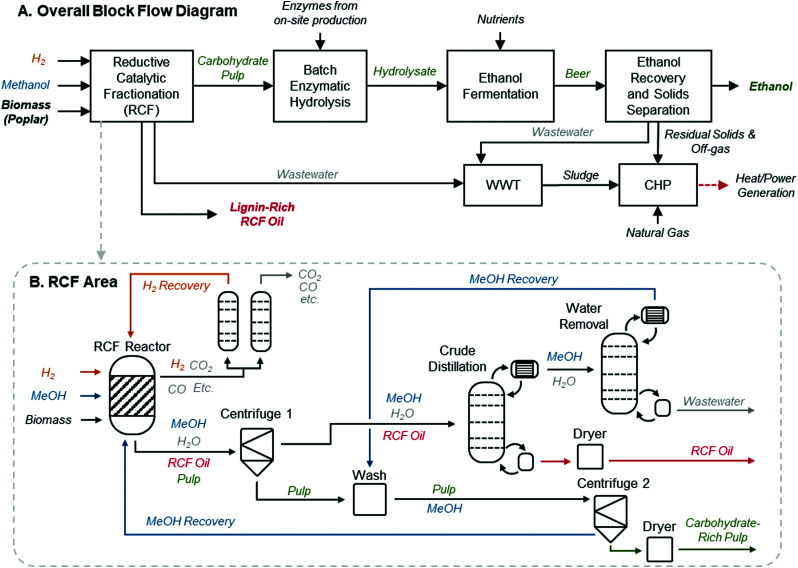
RCF biorefinery design. (A) Block flow diagram of an RCF-based cellulosic ethanol biorefinery. This process follows a configuration similar to that described by Humbird *et al.*, with the dilute-acid pretreatment area replaced with RCF. The RCF area produces a lignin-rich RCF oil as a co-product and a carbohydrate-rich pulp. Carbohydrate pulp, the residual biomass solids after the lignin is removed during RCF, is isolated and saccharified to C_5_ and C_6_ sugars *via* enzymatic hydrolysis, fermented to ethanol, and recovered to produce fuel-grade ethanol. WWT is the wastewater treatment area. CHP is the combined heat and power area where a combination of natural gas, sludge from WWT, and residual solids from ethanol production are burned to generate steam for process heat and electricity. Excess electricity is sold to the grid as a co-product. (B) The process diagram highlighting major unit operations in the RCF area of the biorefinery for the methanol case. The solvent pump-around RCF reactor design is shown in Fig. S2 (ESI†). The gaseous products are sent to a PSA unit to recover H_2_ to be recycled to the reactor, and the liquid and solid products are separated *via* centrifugation to isolate dilute RCF oil, which is concentrated in a crude distillation column, and water is removed from methanol in the water removal distillation column. After washing with methanol to recover additional lignin oil, the carbohydrate-rich pulp is isolated *via* a second centrifuge and the methanol rinse is recycled back to the RCF reactor. Major heating and cooling duties were heat integrated to reduce overall energy demand. A process flow diagram with labeled streams and a corresponding table of stream compositions is included in Fig. S1 and Table S2 (ESI†).

A process model for an RCF-based biorefinery was developed and used as the basis for TEA and LCA. Here we provide an overview of assumptions and process configuration – more detail may be found in Materials and methods. The process model leverages the framework and process assumptions of the cellulosic ethanol model described by Humbird *et al.*, similarly assuming an *n*th-plant design with a 2000 dry metric ton per day feedstock flow rate, and building from an Aspen Plus V10 process simulation and Microsoft Excel economic spreadsheet published by the National Renewable Energy Laboratory (NREL).^46^ The choice to maintain feedstock flow rate consistent with previously published reports provides a direct point of comparison to other biomass valorization TEA models without being confounded by alternative economies of scale. Fig. 1A shows an overview of the major process areas of the lignin-first biorefinery model. Briefly, biomass is delivered to RCF processing for lignin extraction and depolymerization, where RCF replaces the dilute acid pretreatment area present in the original referenced biorefinery model.^46^ In this work, hybrid poplar was used as the feedstock rather than corn stover in the Humbird model.^46^ Poplar has been proposed as an energy crop, as there are fast growing clones that can be cultivated on marginal lands.^47^ Lignin monomer yields from RCF are generally highest for hardwoods, and hardwoods have higher lignin content than herbaceous feedstocks.^48^ Furthermore, more literature data is available on the impact of tuning RCF conditions with poplar than other feedstocks.^34^ The carbohydrate-rich pulp is transferred after the RCF step to enzymatic hydrolysis for saccharification and fermentation to ethanol. Enzymatic hydrolysis has been successfully demonstrated on delignified carbohydrate-rich pulp which had previously undergone RCF,^20^ and RCF hydrolysate has been fermented to ethanol.^28^ Although sugar yields from carbohydrates can vary based on the feedstock used and fractionation conditions, for purposes of this study, enzymatic hydrolysis yields of glucose from residual cellulose are held at 90%, which is supported by evidence collected from bench-scale experiments.^20,28,49^ Due to the removal of the dilute acid pretreatment area initially present in the model, xylan is assumed to be saccharified to xylose at yields of 85% through the addition of xylanases to the saccharification reactor at loadings consistent with the initial model basis of 20 mg per g cellulose. The whole slurry hydrolysate is then fermented to ethanol, followed by ethanol distillation and molecular sieve purification. Residual solids and off-gases downstream of ethanol fermentation are routed to a boiler to be burned along with imported natural gas for process heat and electricity. All other process assumptions downstream of the RCF process area were maintained consistent with those used by Humbird *et al.* (summarized in Materials and methods).^46^

Focusing on the RCF area of the biorefinery, the base-case, referred to hereafter as the “methanol case,” is modeled following common RCF conditions from the literature,^11,14,23^ utilizing imported methanol solvent and hydrogen gas. This case centers many of its operating assumptions on work by Anderson *et al.* with some modification to account for a focus here on future target projections.^29^ The yields and operating conditions used in this case and the other cases *vide infra* are based on laboratory-scale results, but we note that recently-published examples of pilot-scale RCF have shown similar yields in 50 L batch reactors,^32,50^ providing a promising outlook for RCF scale-up. Fig. 1B gives a high-level process flow diagram for this configuration. To capture a generic but promising reactor design that can be informative to many operating modes, we assumed a hybrid batch/flow-through reactor, where the solvent is continuously recycled in a pump-around loop over the duration of the RCF reaction (Fig. S2, ESI†). We hypothesize that this approach will allow for reduced solvent loadings relative to a purely flow-through configuration while eliminating the need for a catalyst-separation step. Poplar (<5 mm, 20% moisture content, complete composition assumptions in ESI,† Table S1) is loaded into one of four ∼600 m^3^ RCF reactors, operationally staggered such that three are operating at any given time and one is being loaded/unloaded, with assumed 1 hour turnaround time per reactor. Each operating reactor is charged with preheated 9 : 1 (vol/vol) methanol : water solvent at a ratio of 9 L per dry kg biomass feed, similar to solvent loadings typically associated with batch operating configurations while maintaining sufficient free solvent availability for the recirculating reactor. The reactor is operated isothermally at 200 °C and 60 bar. Hydrogen gas is continuously fed into the reactor between the biomass and catalyst beds at a rate of 10 L (STP) per minute per dry kg biomass feed. Unreacted hydrogen is recovered *via* an inline flash and pressure swing adsorption (PSA) and recycled back to the process. The catalyst bed is loaded with 15 wt% Ni/C at a ratio of 1 : 10 catalyst : dry biomass feed by weight and assumes annual replacement. A total of 70% biomass delignification is assumed for the reaction, with solubilized lignin weight percent of monomers, dimers, and oligomers of 50%, 25%, and 25%, respectively (see Materials and methods for property estimation of lignin streams). Alcohol reforming to gases was set to 0.5 wt% based on similar observed values from bench-scale experiments, but was assumed also to capture a small but unknown percentage of solvent losses to reaction with carbohydrates, sugars, or acetals.^12^

After a 3 hour reaction time, pressure is reduced to 5 bar, with a flash used to remove the vapor phase and recover excess hydrogen. The product stream is routed to two-stage centrifugation with an intermediate wash step with recovered methanol to improve RCF oil recovery. The separated solids at 30% insoluble solids are dried to recover residual methanol and reduce toxicity effects downstream, then routed to enzymatic hydrolysis to be ultimately converted to fuel-grade ethanol *via* fermentation. The liquid stream is sent to solvent and product recovery. Two distillation columns are employed, the first acting as a “crude” separation step, removing 99.95% of methanol and 96.7% of water, yielding a diluted RCF oil. The distillate is routed to a second column, where water is removed to meet solvent purity requirements of 9 : 1 methanol : water ratio by volume. The 9 : 1 methanol : water volumetric ratio was chosen to maintain high methanol concentrations in the solvent, but reduce column cost and energy requirements that would be necessary to achieve a highly pure solvent typical in laboratory-scale experiments. Removed water is routed to wastewater treatment. To reduce total solvent use in the process, recovered solvent is used in the intermediate wash step during solid/liquid separation and directly routed back to the RCF reactor area. Dilute RCF oil is dried with process heat to contain no more than 0.5 wt% water and subsequently cooled to be sold as the lignin-derived product. The subsequent processing of the RCF oil to a saleable end-product is outside the scope of this analysis. Heat integration was conducted for major process heating and cooling operations, such as cooling of reactor effluent and solvent recovery preheating, with a temperature difference of hot and cold streams of no less than 10 °C, chosen to minimize wasted process utilities while also considering impacts on sizing and costs for heat exchangers. A labeled process flow diagram and a full summary of stream compositions are included in Fig. S1 and Table S2 (ESI†).

Once completed, the material and energy balance details from the methanol-case process model were used to develop facility-level air emission estimates and a life cycle inventory (LCI). The material balance from the process design along with the U.S. Environmental Protection Agency's (EPA) Compilation of Air Pollution Emission Factors Report (AP-42),^51^ EPA guidance documents (*e.g.*, for equipment leak estimation),^52^ and predictive models (*e.g.*, TANKS)^53^ are utilized to estimate emissions of criteria and hazardous air pollutants (HAPs).

The functional unit for the attributional LCA is 1 kg of the lignin fraction of RCF oil. The system boundary is farm-to-gate; upstream processes providing inputs to the biorefinery and the biomass cultivation stage are included in the boundary. Ethanol combustion as biofuel and downstream processing of the lignin fraction are excluded from this LCA, as the objective of this work is to identify RCF process parameters that are highly impactful on LCA metrics. Data for background (upstream of the biorefinery) processes are sourced mainly from the DATASMART life cycle inventory database,^54^ with a poplar farming model from Dunn *et al.*^55^ and additional process data from published studies.^56^ The poplar feedstock is treated as purpose-grown for the RCF biorefinery; although other studies have shown that biorefinery LCA metrics can be improved by using waste feedstocks such as bark and wood chips and forestry residues,^57^ we do not consider these alternatives here.

To align with cost assumptions in the TEA model, methane steam reforming is used for the upstream hydrogen production process. A renewable hydrogen source could also be used; however, the contribution of hydrogen to overall LCA impacts was relatively low, limiting any potential benefit from using renewable hydrogen. For this reason, only hydrogen from methane steam reforming is considered in this analysis. Credits for electricity sales are calculated by assuming that the electricity generated from the boiler steam turbine system (in excess of biorefinery power demands) displaces U.S. grid electricity – this is the system expansion or displacement method. Allocation is then applied to calculate the life cycle impacts of the RCF oil lignin fraction. Two allocation schemes are applied: co-product mass and economic value. The non-lignin fraction of the RCF oil is treated as a waste and is not allocated any life cycle impacts. This results in a conservative estimate of life cycle impacts associated with lignin fraction production from the RCF biorefinery. In the case that the remainder of the RCF oil is also sold as a co-product, life cycle impacts allocated to the lignin fraction and to ethanol will decrease. Unless noted, numerical results reported in the body of this paper are calculated under mass allocation, with economic allocation results given in the ESI.† Mass and economic allocation factors for each RCF case are given in Table S3 (ESI†).

### Identifying cost drivers for the minimum selling price of RCF oil

In this work, the primary economic metric generated from TEA is the minimum selling price of RCF product required to support the sale of the ethanol product at $1.65 per gallon (equal to $2.50 per gallon of gasoline equivalent when normalized to the lower heating value of conventional gasoline – 122.5 MJ per gal),^58^ evaluated by solving a discounted cash flow rate of return for 10% internal rate of return over a 30-year biorefinery lifetime to produce a net present value of zero. The oil produced from RCF is a mixture of both extracted carbohydrates (encompassing sugars, sugar-derived polyols, and soluble oligosaccharides) and the depolymerized lignin fraction consisting of monomers, dimers, and oligomers. While many laboratory studies include liquid–liquid extraction as an additional workup step to further isolate lignin components in the RCF oil from solubilized carbohydrates, for this analysis, we chose to forgo this step to reduce overall heat demand associated with organic phase solvent recovery. More importantly, this approach leaves the analysis agnostic to the potential RCF product options and associated upgrading strategies by treating crude RCF oil as a feedstock for subsequent processing.^59^ Thus, to better understand economics in terms of the components and upgrading strategies, we report three economic metrics:

– MSP-crude RCF oil = the minimum selling price (MSP) of the entire crude RCF product, consisting of both lignin and carbohydrate fractions.

– MSP-lignin fraction = MSP of only the lignin fraction of RCF oil, calculated by normalizing MSP-crude RCF oil by the lignin content of the oil (65%). This metric is applicable if only the lignin fraction will be used for downstream valorization.

– MSP-monomer fraction = the MSP if valorizing only the lignin monomer fraction of RCF oil, *via* normalizing MSP-crude RCF oil by the monomer content (34%).

We also report life cycle metrics using similar nomenclature of GWP-XXX and CED-XXX, where the GWP and CED are normalized to the functional unit of 1 kg of RCF oil lignin fraction. Holding the ethanol selling price at $1.65 per gallon, the MSP-crude RCF oil for the methanol case is $1.13 per kg (see Table S4 (ESI†) for a summary of the process and economic results). Normalizing by lignin and monomer content, the associated MSP-lignin fraction and MSP-monomer fraction values are $1.74 per kg and $3.63 per kg, respectively. Because it summarizes the entirety of the biorefinery capital and operating costs, as well as yields and co-product credits, the minimum selling price (MSP) can serve as a single economic indicator of the feasibility of the process. The MSP also serves as a reference point to ascertain which upgrading strategies and final products are most feasible. For example, a specific co-product with a market value that falls below the MSP is unlikely to be profitable unless process economic improvements can be made.

**Fig. 2:**
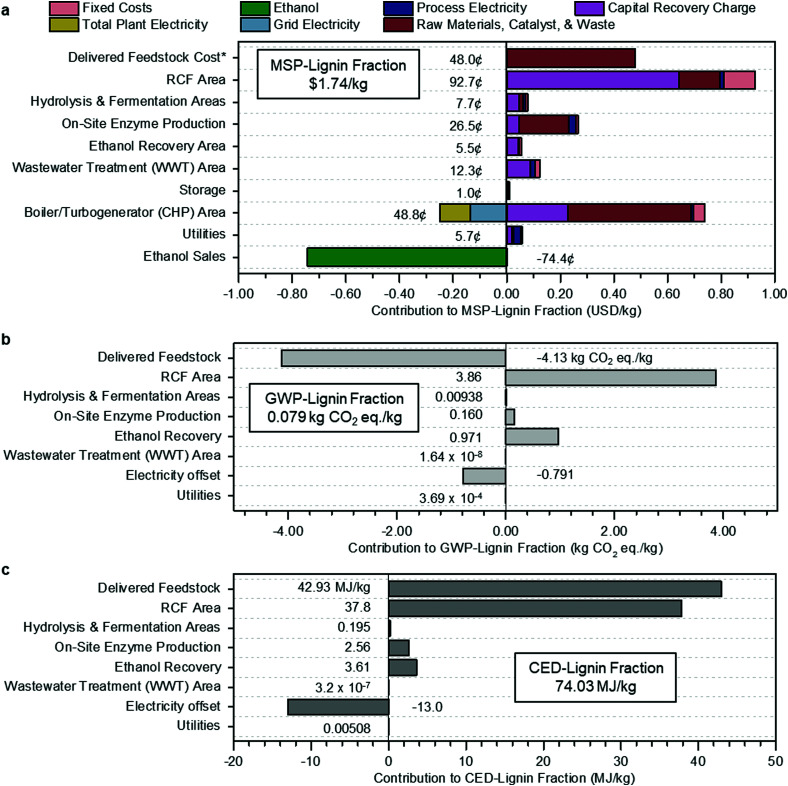
Methanol case economics, GWP, and CED. (a) Contributions to the MSP of the lignin portion of RCF oil (MSP-lignin fraction). Fixed costs include labor and overhead. Grid electricity is the excess generated electricity sold to the grid, process electricity is the electricity requirement for that process area, and total plant electricity is the electricity generated to offset process electricity. Raw materials include biomass feedstock and process inputs such as makeup solvent, hydrogen, glucose for enzyme production, and natural gas. Capital recovery charge accounts for capital depreciation (capital costs), annual income tax, and return on investment. The cost categories on the *y*-axis are organized by process area, corresponding to the block flow diagram in Fig. 1, along with input and output costs such as feedstock, enzyme production, utilities, and ethanol sales that are not encompassed in a specific area. The sum of all individual contributions is the MSP-lignin fraction, or MSP of the lignin portion of the RCF oil product, at $1.74 per kg. *Cost of feedstock includes all upstream feedstock logistics, handling, and pre-processing steps up to the reactor. (b) Breakdown of contributions to GWP-lignin fraction. The results shown are normalized to the production of 1 kg lignin fraction and allocated according to mass. Economic allocation results are given in Table S5 (ESI†). Delivered feedstock includes CO_2_ uptake by growing poplar. The boiler/turbogenerator (CHP) area is not shown in (b) or (c) because impacts for that area have been divided between process areas with steam demand according to the fraction of total steam used by each area: RCF area (89.6%), hydrolysis & fermentation areas (10.2%), and on-site enzyme production (0.2%). Electricity offset is the emissions credit assigned to the biorefinery from displacement of grid electricity. The total GWP, including the electricity offset and CO_2_ uptake by growing poplar, is 0.079 kg CO_2_-eq per kg under mass allocation and 0.131 kg CO_2_-eq per kg under economic allocation. (c) Breakdown of contributions to CED-lignin fraction. Results shown are normalized to 1 kg lignin fraction and allocated according to mass. Economic allocation results are given in Table S6 (ESI†). As was done for GWP, CED for the CHP area was divided between steam-using process areas, and the CHP area is not shown separately in the figure. Electricity offset is the energy credit from displacement of grid electricity. The total CED is 74.03 MJ per kg under mass allocation and 122.04 MJ per kg under economic allocation. All data shown in Fig. 2 are included in numerical form in Table S6 (ESI†) for Fig. 2a and Table S7 (ESI†) for Fig. 2b and c.

The cost breakdown of the RCF oil, expressing costs, and revenues for each process area regarding its contribution to the MSP (MSP-lignin fraction) is summarized in Fig. 2a.

The RCF area of the biorefinery accounts for $0.93 per kg of the MSP-lignin fraction, with a majority of cost in this area from the capital cost of the RCF reactors ($0.57 per kg). Substantial cost is incurred in process heat generation in the boiler/turbogenerator area, with natural gas imports (red bar for raw materials) adding an additional $0.46 per kg while also requiring larger boilers and turbogenerators (purple bar for capital recovery charge) to handle the increased heat demand and generated steam. Costs from this area are offset by $0.13 per kg *via* the generation of excess electricity to be sold to the grid as a co-product. Revenues from ethanol sales further offset the total cost by an additional $0.75 per kg. Total ethanol production (206.5 MML per year) and yield (314.3 L per dry metric ton feedstock) are slightly lower than reported in the Humbird *et al.* design report (230.9 MML per year and 329.6 L per dry metric ton feedstock, respectively), despite increased carbohydrate content in poplar (65.2%) relative to the original corn stover basis (59.0%). These reductions are driven primarily by 10% removal of initial biomass cellulose fraction and 7% removal of initial biomass xylan fraction through the RCF reactor, and a reduced onstream time from 96% to 90% (pertinent only to the annual ethanol production output metric). Feedstock contributions were the third-highest contributor to the total cost, accounting for $0.49 per kg of the MSP-lignin fraction with a feedstock cost of $80 per dry U.S. ton.^60–62^ This feedstock cost includes all upstream feedstock logistics, handling, and pre-processing steps up to the reactor, assuming the poplar was grown for the purpose of biorefining. Other studies have shown that this cost can be reduced through the use of waste feedstocks.^45,63^

The biorefinery GWP and CED for 1 kg lignin fraction under mass allocation are shown in Fig. 2b and c, respectively. The RCF Area is the most significant contributor to life cycle GWP (3.86 kg CO_2_-eq per kg) and the second-largest contributor to CED (37.8 MJ per kg), mainly due to the high heat demand in this area. Delivered Feedstock provides a substantial GWP offset of −4.13 kg CO_2_-eq per kg through carbon uptake by growing biomass but is the largest contributor to CED through material and energy inputs required by the farming process at 42.92 MJ per kg. Excess electricity sold back to the grid also provides a GWP offset of −0.79 kg CO_2_-eq per kg and a CED offset of −13.02 MJ per kg.

**Fig. 3:**
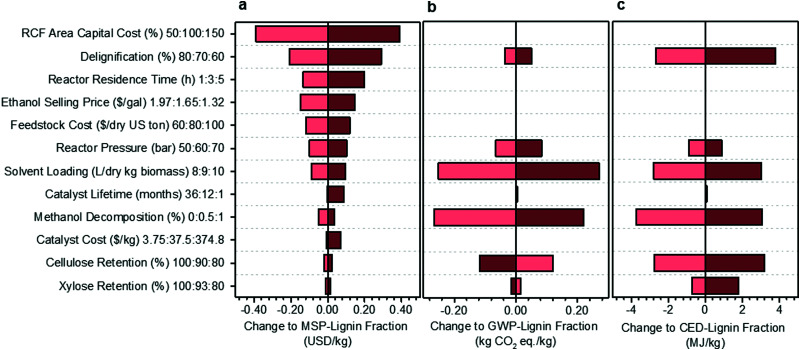
Sensitivity analysis of the methanol case. Results of single point sensitivity analyses for change to the (a) MSP-lignin fraction, or MSP of the lignin constituents in RCF oil (base normalized price = $1.74 per kg), (b) GWP-lignin fraction under mass allocation, and (c) CED-lignin fraction under mass allocation. For each sensitivity case, the key variable was modified to either its minima or maxima while holding all other variables constant to the methanol case. The minima and maxima values used in this analysis are shown in the vertical axis labels as low cost (light red): base: high cost (dark red). Low cost and high cost indicate variable modifications which led to respective net reductions or increases in the MSP-lignin oil. Reasonable minima and maxima were chosen to understand quantitative impacts based on expected uncertainty, prospective process modifications, or potential limits of each variable, and were then used to evaluate the change in MSP-lignin fraction, GWP-lignin fraction, and CED-lignin fraction. Full rationale for the selection of minima and maxima is given in Table S8 (ESI†). All data shown in Fig. 3 is included in numerical form in Table S9 (ESI†). Sensitivity results for change to MSP-crude RCF oil are given in Table S10 (ESI†). Sensitivity results for GWP and CED under economic allocation are given in Table S11 (ESI†).

A univariate sensitivity analysis of the methanol case was conducted to identify which parameters are most impactful for improving the economic feasibility and reducing the environmental impacts of RCF (Fig. 3). It is also helpful for estimating uncertainty in the process model due to lack of data or unforeseen scale-up challenges. This analysis selected several process assumptions to be independently varied to determine their relative impact on overall process economics, expressed here as the MSP of the lignin component of RCF oil (MSP-lignin fraction), and on biorefinery GWP and CED. RCF area capital cost was among the most impactful parameters, with a 50% reduction in RCF capital cost leading to a 22% reduction in MSP-lignin fraction. Impacts of capital cost reduction are mirrored by variables that directly impact RCF reactor sizing and costing, such as residence time, reactor pressure, and solvent loading.

While there will be some energy and heating impacts associated with a shorter residence time, primary cost reductions are realized through the significant decrease in total reactor volume required, leading to an RCF area capital cost reduction of 17% and resultant MSP-lignin fraction reduction of 7.6%. Similarly, pressure effects constitute an important reactor costing consideration, with higher pressures requiring significantly thicker reactor vessel walls and more robust reactor internals. Reducing pressure from 60 bar to 50 bar reduced RCF area capital cost by 11% and resultant MSP-lignin fraction by 5.7%. In contrast, reductions in cellulose retention of 10% only increased the MSP-lignin fraction by 1.4%. Catalyst lifetime and catalyst cost are estimated to exhibit minimal impact on MSP-lignin fraction, even when cost was increased by an order of magnitude (as may be observed when changing metals from nickel to palladium – see Materials and methods for catalyst cost assumptions) and lifetime was reduced to 1 month.

A subset of the variables used to examine cost sensitivity were also used to examine environmental impact sensitivity. Variables with no impact on the life cycle model, such as RCF area capital cost and ethanol selling price, were excluded from the environmental sensitivity study. Solvent loading is highly impactful on both environmental impacts; decreasing the loading from 9 L per dry kg to 8 L per dry kg results in a potential decrease of 317% (from 0.079 to −0.17 kg CO_2_-eq per kg) in GWP and 15% in CED. Reducing the methanol decomposition from 0.5% to 0% also offers significant impact reductions of 335% (from 0.079 to −0.187 kg CO_2_-eq per kg) in GWP and 5% in CED. Cellulose and xylose retention were the only parameters that caused opposite effects on GWP and CED. Increasing cellulose retention increased GWP by 150% while decreasing CED by 4%, and increasing xylose retention increased GWP by 19% while decreasing CED by 1%. The increasing GWP with increased carbohydrate retention results from additional process-level, non-fossil CO_2_ emissions from fermentation, while the CED decreases are primarily due to changes in lignin fraction yield and in material and energy input requirements.

The single-variable sensitivity analysis in Fig. 3 suggests that percent delignification is a crucial economic and moderately important environmental consideration. However, this single-variable analysis is an over-simplification because delignification is a function of operating conditions, such as residence time, reactor pressure, and solvent loading (each evaluated as its own variable). To quantify tradeoffs between reducing production costs at the expense of percent delignification, we reduced residence time, pressure, or solvent loading and calculated how much degree of delignification could be reduced while still maintaining an identical MSP-lignin fraction of $1.74 per kg (Table 1). When residence time was shortened from 3 hours to 1 hour, up to a 7% reduction in delignification (to 64%) could occur while maintaining consistent economics. A 6% reduction (to 66%) in delignification could be accommodated when reactor pressure was reduced to 50 bar or solvent loading decreased to 8 L per dry kg biomass. When all three conditions were combined simultaneously, total delignification could be reduced by up to 17% (to 58%). Combining the three conditions also leads to GWP being reduced by 368% to −0.212 kg CO_2_-eq per kg, and CED being increased by 0.32% to 74.27 MJ per kg. We note that tuning conditions such as residence time and reactor pressure could also influence relative proportions of lignin monomers, dimers, and oligomers. For purposes of this analysis, product distributions were assumed constant to the methanol-case, and further optimizations would be required if a specific RCF oil fraction is the target product.

**Table 1:** Analysis of trade-offs between delignification and residence time, reactor pressure, and solvent loading. This analysis modified the process variables and calculated the allowable reduction in delignification to maintain an MSP-lignin fraction identical to the base case. GWP-lignin fraction and CED-lignin fraction were then calculated based on the adjusted operating conditions. The top portion of the table shows the inputs of residence time, reactor pressure, and solvent loading. All other variables were held constant to the methanol case. The bottom portion of the table shows the delignification percent that is necessary to maintain the MSP-lignin fraction of $1.74 per kg, along with the GWP-lignin fraction and CED-lignin fraction (under mass allocation) that were calculated for each set of inputs

	Base	Reduce residence time	Reduce pressure	Reduce solvent loading	Reduce all 3
Residence time	3 h	1 h	3 h	3 h	1 h
Reactor pressure	60 bar	60 bar	50 bar	60 bar	50 bar
Solvent loading (L per dry kg biomass)	9 L kg^−1^	9 L kg^−1^	9 L kg^−1^	8 L kg^−1^	8 L kg^−1^
Calculated delignification to maintain MSP-lignin fraction of $0.79 per kg	70%	64%	66%	66%	58%
GWP-lignin fraction (kg CO_2_-eq per kg)	0.079	0.095	−0.160	0.031	−0.212
CED-lignin fraction (MJ per kg)	74.03	75.79	72.46	74.67	74.27

**Fig. 4:**
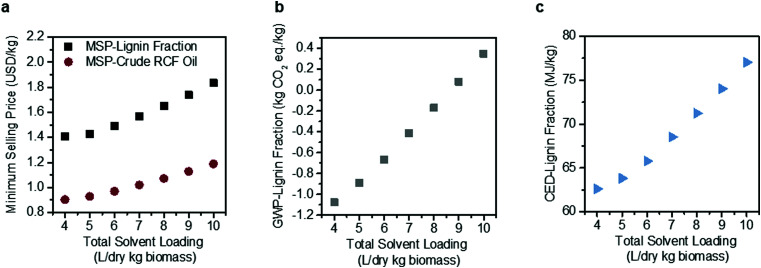
Sensitivity analysis on methanol solvent loading. Effects of solvent loading on (a) MSP-crude RCF oil and MSP-lignin fraction, (b) GWP-lignin fraction under mass allocation, and (c) CED-lignin fraction under mass allocation. Solvent loading was varied from 4–10 L per dry kg biomass holding all other process assumptions constant. The methanol-case solvent loading is 9 L per dry kg biomass. Negative GWP indicates the potential for greater GHG uptake than emissions over the lignin fraction life cycle. All data shown in Fig. 4 are included in numerical form in Table S12 (ESI†). The sensitivity analysis results for GWP and CED under economic allocation are given in Table S13 (ESI†).

Solvent loading exhibits a compounding effect on process economics, impacting both reactor size due to volumetric throughputs and process heat required for reactor pre-heating and solvent recovery, thus also warranting a more detailed sensitivity analysis (Fig. 4). Under the methanol case, total RCF area heat demand is 295 MW, which is equivalent to 72.8% of the energy content of the biomass itself when normalized to the Aspen-calculated lower heating value (LHV) of the feedstock (406 MW, 0.0732 MJ per dry kg). The model estimates that 56% of the RCF area heat demand is for solvent recovery, either through distillation or drying of the pulp. Energy demand may be reduced as solvent loading decreases, reaching 191.8 MW, or 41% of the energy content of the biomass at a total solvent loading of 4 L per dry kg biomass.

Decreasing solvent loading to 4 L per dry kg biomass also reduces the GWP from 0.079 kg CO_2_-eq per kg to −1.078 kg CO_2_-eq per kg, with a negative number indicating that carbon dioxide uptake by growing poplar is greater than carbon dioxide and other greenhouse gas emissions elsewhere within the biorefinery life cycle. The CED likewise decreases by 15% when solvent loading is reduced, from 74.03 MJ per kg at 9 L per dry kg to 62.61 MJ per kg at 4 L per dry kg.

### Benchmarking other process designs relative to the methanol case

**Fig. 5:**
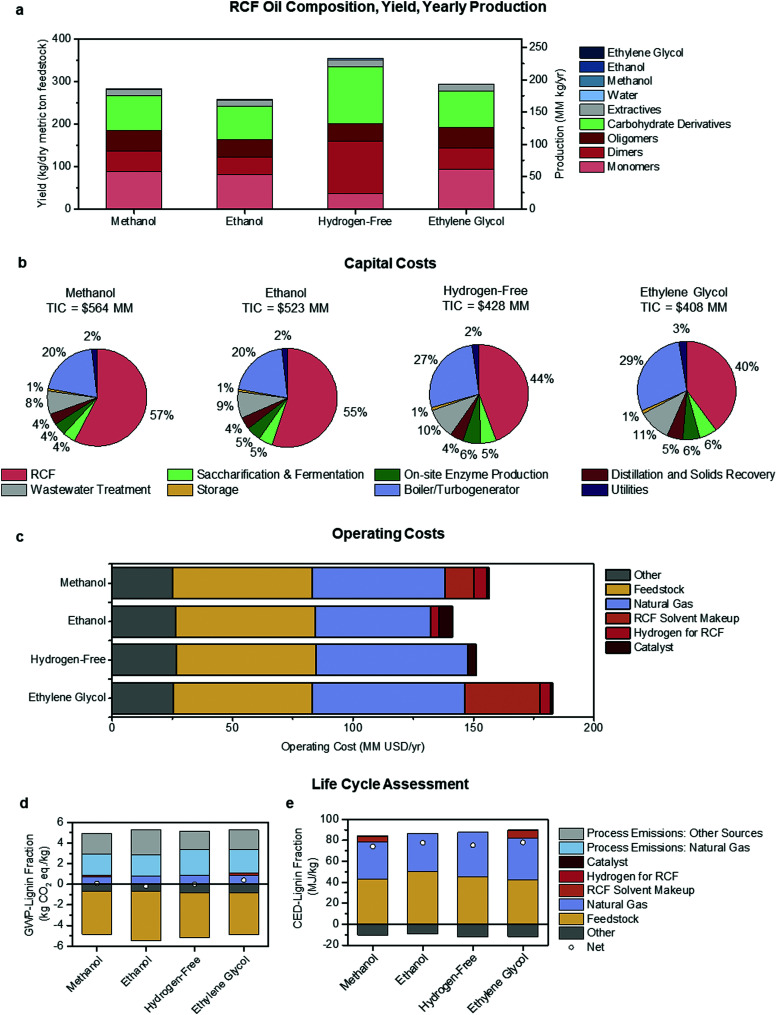
Economic and environmental comparison of RCF process configurations. Methanol utilizes methanol solvent and hydrogen gas, ethanol uses solvent generated in the biorefinery, hydrogen-free generates hydrogen *in situ* from hemicellulose, and ethylene glycol uses hydrogen gas and ethylene glycol solvent. (a) A summary of RCF oil composition, yield, and productivity. The composition was based on literature values for similar processes at the bench-scale. Yield and productivity values were outputs of the process model at a 2000 dry metric tons feedstock/day throughput. (b) Capital cost breakdown by area for each process configuration, with the total installed capital cost (TIC) included above the pie charts. (c) Yearly operating costs for each process configuration. “Other” costs are associated with carbohydrate conversion to ethanol downstream of the RCF area. (d) GWP-lignin fraction for each configuration shows categories that contribute to carbon emission and carbon uptake, with net GWP-lignin fraction indicated with a circle. (e) CED-lignin fraction for each configuration shows categories that contribute to energy consumption and energy generation (in the form of excess electricity sold to the grid), with net CED-lignin fraction indicated with a circle. See Fig. 1 and Fig. S3–S5 (ESI†) for diagrams of each configuration, Table 2 for a complete list of assumptions for each case, and Tables S17–S21 (ESI†) for full tabulated data shown in this figure. GWP-lignin fraction and CED-lignin fraction under economic allocation for each configuration are given in Table S22 (ESI†).

Three additional process scenarios were analyzed to understand better the complexities and tradeoffs associated with changing process designs: the ethanol case, the hydrogen-free case, and the ethylene glycol case (descriptions *vide infra*). Because the reagent composition and reaction conditions ultimately determine the delignification extent, carbohydrate retention, and lignin monomer yields from the RCF step, we based the models directly on experimental data in the literature on RCF of hardwood feedstocks. As a result, each configuration utilized a slightly different set of assumptions summarized in Table 2. The modified process flow diagrams are included in Fig. S3–S5 (ESI†). The results of the TEA and LCA of each case are summarized in Fig. 5 and Table 3, with economic case summaries in Tables S14–S16 (ESI†). While these are only three of many proposed sets of operating conditions, they represent an acceptable range of conditions to provide broader design insights.

**Table 2:** Operating assumptions for four RCF biorefinery designs. Key operating assumptions for each process design sensitivity case. These assumptions are based on the reported experimental conditions and yields from Anderson *et al.*^29^ for the methanol case, Renders *et al.*^18^ for the ethanol case, Galkin *et al.*^21^ for the hydrogen-free case, and Schutyser *et al.*^17^ for the ethylene glycol case. The reactor pressure was based on the vapor pressure of the solvent composition at the reactor temperature. The reactor temperature, residence time, catalyst composition, delignification, lignin composition, and carbohydrate retention were based on each case's bench-scale literature data. Alcohol reforming to gases was set to 0.5 wt% based on similar observed values from bench scale experiments^12^

	Methanol	Ethanol	Hydrogen-free	Ethylene glycol
RCF reactor solvent (volumetric ratio)	9 : 1 methanol : water	85 : 15 ethanol : water	1 : 1 ethanol : water	99 : 1 ethylene glycol : water
Solvent loading (L per dry kg biomass feed)	9	9	9	9
Hydrogen loading (L_STP_ per min per dry kg biomass)	10	10	0	10
RCF reactor temperature (°C)	200	200	210	200
RCF reactor pressure (bar)	60	50	30	6
RCF reactor residence time (h)	3	3	2	3

Catalyst	15 wt% Ni/C	5 wt% Pd/C	5 wt% Pd/C	15 wt% Ni/C

Biomass delignification (wt%)	70%	60%	75%	70%
Solubilized lignin composition (wt%)				
Monomers	50%	50%	20%	50%
Dimers	25%	25%	60%	25%
Oligomers	25%	25%	20%	25%
S-Monomer composition (wt%)				
4-Propylsyringol	75%	20%	75%	75%
Dihydrosinapyl alcohol	25%	80%	25%	25%
G-Monomer composition (wt%)				
4-Propylguaiacol	66%	5%	66%	66%
Dihydroconiferyl alcohol	34%	95%	34%	34%

Carbohydrate retention (wt%)				
Cellulose	90%	95%	97%	90%
Xylan	93%	70%	38%	93%
Arabinan	40%	70%	38%	40%
Galactan	50%	70%	38%	50%
Mannan	50%	70%	38%	50%

Alcohol reforming to gases (wt% of alcohols)	0.50%	0.50%	0.50%	0.50%

**Table 3:** Economic, production, and sustainability metrics. Minimum selling prices are shown in terms of the whole crude RCF oil (containing both lignin and extracted polysaccharides), normalized to the lignin component of RCF oil, and normalized to the lignin monomer content in the oil. GWP and CED are shown calculated from mass allocation and normalized to the lignin component of RCF oil

	Methanol	Ethanol	Hydrogen-free	Ethylene glycol
Ethanol yield	314.3 L per dry metric ton	263.2 L per dry metric ton	265.5 L per dry metric ton	314.0 L per dry metric ton
MSP-crude RCF oil	$1.12 per kg	$1.18 per kg	$0.76 per kg	$0.98 per kg
MSP-lignin fraction	$1.74 per kg	$1.88 per kg	$1.34 per kg	$1.51 per kg
MSP-monomer fraction	$3.63 per kg	$3.76 per kg	$7.58 per kg	$3.07 per kg
GWP-lignin fraction	0.079 kg CO_2_ eq. per kg	−0.175 kg CO_2_ eq. per kg	−0.018 kg CO_2_ eq. per kg	0.392 kg CO_2_ eq. per kg
CED-lignin fraction	74.03 MJ per kg	77.47 MJ per kg	75.36 MJ per kg	77.93 MJ per kg

In our analysis, the biorefinery produces ethanol from the carbohydrate-rich pulp and therefore could eliminate the need for imported methanol by diverting part of the ethanol product to be used as the solvent in the RCF operation. The ethanol case is similar in configuration to the methanol case and bases many of its assumptions on work by Renders *et al.*^18^ Solvent purity requirements, in this case, are 85 : 15 ethanol : water by volume, which was chosen to minimize energy costs in distillation when approaching the ethanol/water azeotrope but maintain sufficient carbohydrate retention in avoiding high water ratios. Given the lower vapor pressure of ethanol and water, the reactor pressure is slightly reduced to 50 bar. Delignification is set to 60% at a 3 hour residence time, based on the literature report.^18^ Hydrogen use and catalyst loadings are also assumed to be consistent to the methanol case with the catalyst changed to 5 wt% Pd/C. Monomer composition and carbohydrate retention is set to be similar to those observed in bench-scale experiments.^18^ Since ethanol is used instead of methanol, purge streams high in ethanol are sent to either downstream distillation in the ethanol beer column or to the vent scrubber, rather than wastewater treatment, or a boiler, making use of existing process equipment and improving overall solvent recovery.

The hydrogen-free case, basing many of its assumptions on work by Galkin *et al.*, provides an assessment of the avoidance of purchased hydrogen (from fossil sources) by generating the necessary hydrogen *in situ*.^21^ Rather than hydrogen gas, solubilized hemicellulose serves as a hydrogen donor for transfer hydrogenolysis to lignin. In this configuration, a 1 : 1 volumetric ratio of ethanol : water is applied, using the ethanol produced from the carbohydrate train as the RCF solvent. Unlike the methanol or ethanol cases, hydrogen gas and subsequent hydrogen recovery equipment are no longer needed and are removed. Reactor pressure is reduced to 30 bar due to a lower saturation pressure of the solvent. Additionally, relatively low ethanol purity requirements avoid the need for a second distillation column. Similar to the ethanol case, a 5 wt% Pd/C catalyst is used, which in this case performs both hydrogenolysis and hydrogen transfer reactions, and purge and wastewater streams are routed to either the ethanol vent scrubber or the beer column. While lower monomer yields were observed by Galkin *et al.* when poplar was used, higher yields were observed in birch.^21^ Yields and product distribution were based on results observed for poplar at a 2 hour residence time.

The ethylene glycol case, basing many of its assumptions on work by Schutyser *et al.*,^17^ replaces the solvent in the methanol-case with ethylene glycol, a solvent that has shown equivalent monomer yields and delignification compared to methanol. However, due to the lower vapor pressure of ethylene glycol, overall reactor pressure is reduced substantially from 60 bar to 6 bar while still maintaining a reactor temperature of 200 °C and solvent in the liquid phase. To capitalize on the lower vapor pressure of the solvent, the continuously recycled flow-through reactor modeled in this study allows hydrogen to be fed at 6 bar while maintaining a ratio of 10 L (STP) per minute per dry kg biomass feed, equivalent to the methanol case. This translates to a lower H_2_ pressure than has been utilized experimentally to date (*i.e.* charging a batch reactor with 30 bar H_2_). It will be important to verify the efficacy of these conditions with ethylene glycol under this configuration. The low volatility of ethylene glycol warranted the design of a new three-column distillation for solvent recovery after pulp centrifugation. The first column operates at a pressure of 1.2 atm and removes 99% of the water at a minimal loss of ethylene glycol (<0.05%). The second column operates under a vacuum (0.2 atm), removing 99.7% of the remaining ethylene glycol while the third and final column operates under a stronger vacuum (0.1 atm) and further removes 95% of residual ethylene glycol from RCF oil for a net solvent recovery of >99.9% across the column network. It has been shown previously that ethylene glycol maintains similar degrees of delignification and carbohydrate retention to that of methanol, and thus these assumptions are assumed to be identical to that of the methanol case.^17^ The use of vacuum distillation is required to avoid more expensive utilities such as high-pressure steam, fired heat, or hot oil systems.

Annual production, yield, and composition of the RCF oil products from each case are summarized in Fig. 5a. Rigorous kinetics of the reactions occurring during RCF was not considered, and it is not the intention of this study to provide a predictive model. Instead, these metrics and distributions were determined *a priori* using information from the literature extrapolated to the modeled biorefinery scale. RCF oil production ranged from 170 MM kg per year to 233 MM kg per year, while crude RCF oil yields ranged between 258 and 354 kg per dry metric ton feedstock in the ethanol and hydrogen-free cases, respectively. However, despite higher quantities of crude RCF oil, the hydrogen-free case produced the lowest amount of lignin monomers at 23.4 MM kg per year and yields of 35.6 kg per dry metric ton feedstock, less than half of those observed in the methanol and ethanol cases and has a lower overall lignin weight percent (Fig. 5a, see Table S17 for numerical composition, ESI†). The methanol case produced the highest quantities of monomers with yields of 88.4 kg per dry metric ton feedstock, or 8.8% mass yields. Fig. 5b shows the distribution of the total installed capital (TIC) (see ESI,† Table S18 for numerical values). The methanol and ethanol cases had the highest TIC at $564 MM and $523 MM, respectively, with capital costs for the RCF process operations making up more than half of the biorefinery total. The hydrogen-free case had a lower TIC of $428 MM and the ethylene glycol case reflected the lowest at $408 MM. The cost of reactor capital primarily drives equipment costs in the RCF area. For example, the total reactor volume required for the methanol case exceeds 1500 m^3^, with thick walls to accommodate high operating pressures and robust reactor internals, resulting in an installed cost of RCF reactors of $290 MM. The other central area of capital expenditure is that of the boiler/turbogenerator for heat and power generation at a total installed cost of $114 MM, needed to meet heat demands for solvent recovery and reactor preheating. Increases in turbine size due to additional steam generation also contribute significantly to capital costs in this area. Despite multiple competing factors, generally, there is a trend that lower pressure and lower residence time during RCF results in lower capital costs.

The operating costs were binned into six major categories and summarized in Fig. 5c. Feedstock and “Other” costs (associated with downstream carbohydrate conversion) were largely agnostic to RCF design at $57.9 MM per year and between $25.2 MM per year and $26.9 MM per year, respectively. The feedstock cost, including all upstream feedstock logistics, handling, and pre-processing steps up to the reactor, was held constant to $80 per dry U.S. ton ($88.2 per dry metric ton) for each process configuration.^60–62^ The differences in process design are highlighted in makeup solvent, natural gas, hydrogen gas, and catalyst costs. As distillation is the primary method for solvent recovery and purification, natural gas operating costs approximately trend with heats of vaporization for each solvent employed. The ethanol case is the lowest at $48.0 MM per year and the ethylene glycol case the highest at $63.0 MM per year. Makeup solvent is required due to entrainment in the carbohydrate pulp, purge streams, and wastewater streams. However, greater than 90% of makeup demand is due to the decomposition/reforming of solvent through the RCF reactor. While this number is seemingly small (set as 0.5% in all cases), given the high total volume of solvent required, this number compounds to contribute $12.1 MM per year in operating costs in the methanol case and up to $31.3 MM per year in the ethylene glycol case with the discrepancy in cost due primarily to the higher cost of ethylene glycol ($0.82 per kg) relative to methanol ($0.29 per kg). Note that 0.5% solvent loss during the reaction was assumed for each design case based on a literature report which showed 0.21 mol% loss of methanol into carbonaceous gasses during a 3 h reaction at 250 °C, utilizing 5 wt% Ru/C and 30 bar H_2_.^12^ We rounded up to 0.5% to account for other possible solvent losses, such as reaction with the sugars or acetyl-groups, but further research is necessary to determine the exact fate of solvent and the dependence of solvent decomposition on reaction conditions and catalyst formulation. Additional information on operating costs is given in Materials and methods. The ethanol and hydrogen-free cases do not include makeup solvent as an operating cost, as the makeup solvent for these cases is sourced internally with a penalty in the observed lower apparent yields of ethanol. Hydrogen gas was a relatively low contributor to overall operating cost at a maximum of $5.5 MM per year in the methanol case. Annual costs associated with catalyst replacement were $0.9 MM per year in the methanol and ethylene glycol cases, where 15 wt% Ni/C was used, and $5.6 MM per year and $3.7 MM per year in the ethanol and hydrogen-free cases, respectively, where 5 wt% Pd/C was modeled.

GWP- and CED-lignin fraction were similarly binned into six process input categories, plus two process-level emissions categories for GWP, and are shown under mass allocation in Fig. 5d and e, respectively. Because both life cycle impacts include positive and negative contributions, the net impact is marked for each case with a circle. The ethylene glycol case exhibits the highest GWP at 0.39 kg CO_2_-eq per kg, due to increased natural gas consumption. Both the ethanol and hydrogen-free cases improved on the methanol case, with GWPs of −0.18 and −0.018 kg CO_2_-eq per kg, respectively. The GWP improvements in both cases were enabled by the avoidance of an externally purchased solvent. Across all four cases, the significant positive contributions to GWP were process-level CO_2_ emissions from natural gas combustion for steam generation and the fermentation step, and the negative GWP contribution consisted of carbon uptake in growing biomass and offsets from electricity sales.

As shown in Fig. 5e, CED-lignin fraction increased slightly over the methanol case for all three alternate process designs. The hydrogen-free and ethylene glycol cases exhibit higher CEDs than the methanol case by 2% and 5%, respectively. Both cases had an increased lignin fraction yield which lowered the CED contribution from feedstock production and increased the CED offset from excess electricity sales; however, both cases also required increased natural gas consumption which more than offset the CED improvements from increased yields. The ethanol and hydrogen-free cases offer GWP benefits but CED penalties relative to the methanol case, due to the interactions between lignin fraction yield and feedstock production impacts. The ethanol case has a lower lignin fraction yield (0.16 kg per dry kg feedstock) compared to the methanol case (0.18 kg per dry kg feedstock), while the hydrogen-free yield is higher (0.20 kg per dry kg feedstock). Feedstock production provides a GWP offset along with a positive CED contribution, which means that improving the lignin fraction yield per dry ton of feedstock will have opposite effects on GWP and on CED. Mitigating this trade-off would require lowering the feedstock production CED while maintaining or improving the carbon sink, which could potentially be accomplished with less intensive farming practices.

The key economic and life-cycle metrics and ethanol yields are summarized in Table 3. The ethanol and hydrogen-free cases show reductions in ethanol yield at 263.2 and 265.5 L per dry metric ton feedstock, respectively, relative to the methanol and ethylene glycol cases at 314.3 L per dry metric ton feedstock. The reduction is primarily due to the utilization of ethanol as a solvent, which in the ethanol case results in losses of 39.2 L per dry metric ton feedstock from alcohol reforming during RCF and losses of 7.5 L per dry metric ton feedstock associated with process purge streams and unrecovered ethanol remaining in wastewater streams. Additionally, the higher water content in the hydrogen-free case leads to increased polysaccharide solubilization during RCF (seen in the more significant fraction of carbohydrate derivatives in the crude RCF oil), which reduced ethanol yields.^9,64^ From Table 3, the hydrogen-free case is estimated to exhibit the lowest MSP-crude RCF oil and MSP-lignin fraction of $0.76 per kg and $1.34 per kg, followed by ethylene glycol at $0.98 per kg and $1.51 per kg, respectively. However, the ethylene glycol case had the lowest MSP-monomer fraction at $3.07 per kg, and the hydrogen-free case is predicted to incur the highest MSP-monomer fraction at $7.58 per kg, due to a comparatively lower yield of monomers in the RCF oil for this case.

### Evaluating a hypothetical membrane separation

In the most promising cases from an economic perspective, ethylene glycol and hydrogen-free, the energy demands associated with solvent recovery amount to greater than 75% of the energy content of the biomass itself, based on its lower heating value. Separations that do not employ a phase change, such as a membrane system, may be able to help alleviate energy and sustainability concerns while simultaneously improving process economics. While there is some energy demand associated with preheating the RCF reactor, 83% of heat demand is attributed to the distillation of the recovered solvent in the ethylene glycol case. Membranes have been demonstrated for the purification of technical lignins,^65^ the isolation of individual components from lignin streams,^66,67^ and for the recovery of organic solvents from other non-lignin (*e.g.*, pharmaceutical) processes.^68,69^ Recently, membrane separations were demonstrated for isolating fractions of reductively depolymerized lignin from solvents,^70^ and liquid-phase separations have been highlighted as a possible approach toward reducing energy consumption during solvent recovery with RCF.^71^ Despite these promising preliminary studies, currently the technology is too premature for us to accurately estimate the cost and life cycle metrics for membrane separations to directly compare with the scenarios in the previous section. However, to evaluate the potential of membrane separations, we developed a hypothetical scenario to determine break-even costs with the use of membranes.

**Fig. 6:**
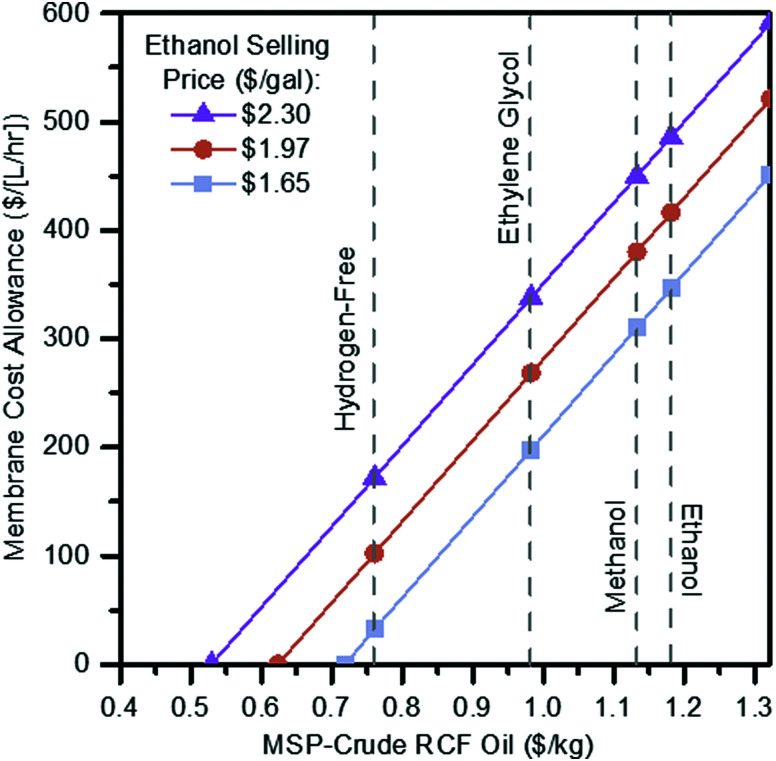
Membrane cost allowance. Sensitivity scan of maximum membrane capital cost allowance per throughput volume in order to achieve a given RCF oil MSP at the fuel selling price target. See Table S23 (ESI†) for tabulated data shown in this figure.

To evaluate potential cost implications, the vacuum distillation step in the ethylene glycol case was removed and replaced with a block representing membrane separations where a majority (99%) of ethylene glycol is assumed to be removed while 95% of the RCF oil and other soluble components move to final vacuum distillation to recover the remaining ethylene glycol (Fig. S6, ESI†). The solvent from both the membrane (containing 5% of the incoming lignin oil) and residual solvent recovered from distillation is recycled back to RCF. Costs for membrane separation of RCF oil from ethylene glycol solvent are primarily a function of membrane price and flux throughput, both of which are dependent on the type, material, and performance of the membrane. Rather than estimating the cost of a specific membrane system, this scenario is evaluated as a sensitivity analysis over a range of costs translated to a $ per [L per h] basis, representing the combination of membrane unit cost ($ per m^2^) and flux (L per m^2^ per h) to provide a starting frame of reference as to maximum allowable costs to break even or improve upon the ethylene glycol case relative to distillation in the present process context. In addition to this approach of evaluating placeholder capital costs for the membrane step, a maintenance charge of 6% per year applied to the overall membrane module is also included as an estimate to represent membrane maintenance and replacement costs. A 6% maintenance factor was assumed for preliminary TEA purposes, representing a two-fold increase over standard 3% maintenance costs applied to all other installed equipment elsewhere. In contrast, the maintenance factor would be specific to a particular membrane type, material, and processing service, the 6% value is assumed as may reflect an average between lower maintenance for the membrane housing and higher for periodic replacement of the membrane itself. With this approach, the total allowance of installed cost for the membrane system to maintain an identical MSP-crude RCF oil was $144 MM. The results of the membrane unit sensitivity scan are shown in Fig. 6, plotted as a maximum allowable membrane capital cost per throughput volume as a function of lignin oil MSP and ethanol selling price goals, and overlaid with break-even points for the ethanol, methanol, ethylene glycol, and hydrogen-free cases ($1.18 per kg, $1.13 per kg, $0.98 per kg, and $0.76 per kg MSP-crude RCF oil, respectively).

For example, in the ethylene glycol case, switching from distillation to membrane recovery of the solvent results in cost reductions (*i.e.* lower required crude RCF oil selling price) at a membrane cost of $198 per [L per h] or less. Additionally, with the elimination of vacuum distillation, the RCF area heat demand is reduced by 66% from 317 MW (78.2% of input biomass LHV) where distillation is used to 108 MW (26.7% of input biomass LHV) where a membrane system is used. This leads to declines in natural gas usage by 80% from 30 600 kg per h to 6100 kg per h, leading the overall lignin fraction CED to decline by 24% from 77.93 MJ per kg to 58.89 MJ per kg.

### Estimation of non-GHG air pollutant emissions

**Fig. 7:**
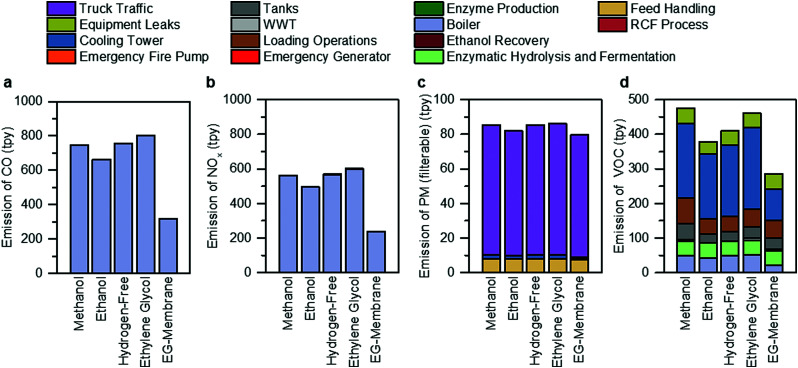
Non-GHG air pollutant emissions. Overview of (a) CO, (b) NO_*X*_, (c) PM, and (d) VOCs for each of the RCF process design cases and the hypothetical membrane separation case, broken down by the source of the emission. The assumptions and methods used for this analysis are summarized in Materials and methods. The raw tabulated data is included in Tables S24–S28 (ESI†). Bar charts for other air pollutants analyzed in this study (PM10, PM2.5, SO_2_, and hazardous air pollutants) are included in Fig. S7 (ESI†).

The facility-level emissions of selected criteria air pollutants (carbon monoxide (CO), nitrogen oxides (NO_*x*_), particulate matter (PM), and volatile organic compounds (VOCs)), in tons per year (tpy), are shown in Fig. 7 and Tables S24–S28 (ESI†) for all RCF cases. Steam generation by the boiler accounts for 99.9% of CO and NOx emissions, while truck traffic and cooling towers are the single largest contributor of PM (41–88%) and VOC (49–51%) emissions, respectively, across all process designs. The ethylene glycol case exhibits the highest CO (601 tpy) and NO_*x*_ (801 tpy) emissions due to high natural gas consumption in the boiler. The ethylene glycol case also contributes to the highest filterable PM emissions (86 tpy) among all process designs, mainly from track traffic, due to the transport of solvents used as inputs by the process. The methanol case has higher VOC emissions (474 tpy) than other alternative process designs due to increased emissions of the use of a more volatile solvent (methanol, vapor pressure of 0.17 bar at 25 °C) from storage and loading operations.

As shown in Fig. 7, the hypothetical ethylene glycol membrane (EG-membrane) case exhibits the lowest emissions across all the designs. Compared to the ethylene glycol case, there is a 60% reduction in facility-level CO and NO_x_ emissions for the EG-membrane design due to low natural gas consumption. In addition, the emissions of filterable PM are reduced by 8% compared to ethylene glycol case due to lower inputs of various chemicals or solvents. VOC emissions are also reduced by 38%, primarily due to a 61% lower cooling tower circulation rate. The emission estimates of other criteria air pollutants are summarized in Fig. S7 (ESI†), and HAPs are presented in Tables S29–S33 (ESI†).

## Discussion, recommendations, and conclusions

In this work, we have identified the primary cost drivers and sustainability considerations for several potential process configurations of an ethanol biorefinery incorporating RCF. While there remain advances to be realized in scaling up the RCF process, we can make several recommendations based on the results of this study for future research and optimization.

Capital expense was the most significant cost driver in the sensitivity analysis on the methanol case, and the lowest capital processes (ethylene glycol and hydrogen-free) exhibited the lowest MSP-crude RCF oil and MSP-lignin fraction. A significant contributor to capital expenses was sizing and costing the RCF reactors, driven by solvent volumes, residence times, and operating pressure. The reduced TIC for ethylene glycol and hydrogen-free cases are mainly driven by the lower RCF operating pressure and lower residence time in hydrogen-free. Thus, we recommend the development of processes that continue to reduce operating pressure and reactor volume. This could include, but is not limited to, reducing solvent loading, optimizing reactor configuration, or reducing solvent vapor pressure. A recent publication of ambient pressure RCF provides an optimistic outlook in this direction.^72^

Solvent loading stood out as a factor that was impactful for MSP, GWP, and CED. In addition to the capital cost required for large reactor volumes, high solvent loadings led to an RCF area energy demand of 72.8% of the heating value of the biomass itself in the methanol case, with 56% of that energy required for solvent recycle alone. When ethylene glycol was used instead of methanol, the CED-lignin fraction increased by 3.89 MJ per kg (5%) due to the energy-intensive distillation of the high-boiling ethylene glycol solvent. The high energy demand coupled with reduced lignin available to burn for process heat led to high natural gas imports (30 600 kg per h for the ethylene glycol case), raising operating costs and negatively impacting process sustainability. Given the significant impact of the solvent on process economics and sustainability, we recommend developing processes that reduce solvent loading and technologies for liquid-phase solvent recycling. Reducing solvent loading in the methanol case from 9 L per dry kg biomass to 4 L per dry kg biomass reduces the GWP from 0.079 kg CO_2_-eq per kg to −1.078 kg CO_2_-eq per kg lignin fraction and reduces the CED by 15%, from 74.03 MJ per kg to 62.61 MJ per kg. Processability challenges may exist at 4 L per dry kg biomass. The total solids fraction of 26% after the reaction leads to concerns about pumpability, ease of conveyance, mass transfer limitations in lignin extraction, and increasing entrainment of solubilized components in the carbohydrate pulp after centrifugation. This motivates additional research in reaction engineering, reactor design, and process design for solvent minimization in lignin extraction.

Our analysis showed that a membrane separation step could eliminate the energy required for distillation and would be economically advantageous at a membrane cost of $198 per [L per h] or less in the ethylene glycol case. To provide an estimate of membrane cost and gauge feasibility, we reference an economic analysis performed by Sultan *et al.*^70^ on a membrane purification of catalytic upstream biorefining (CUB) oil, which is a similar substrate to RCF oil. They estimate the cost of their best-performing polyimide organic solvent nanofiltration (OSN) membrane, Puramem®, at $500 per m^2^. They showed experimental permeance of 0.21 L per m^2^ per h^1^ per bar^1^, and noted this performance is not economically viable. However, they noted that values above 5 L per m^2^ per h^1^ per bar^1^ have been shown in the literature with other solvent separations^73^ and highlighted this as a reasonable target to achieve economic feasibility. Indeed, using their estimate of $500 per m^2^ and target permeability of 5 L per m^2^ per h^1^ (at 1 bar), we calculate $100 per [L per h], which is economically beneficial *versus* distillation in nearly all cases shown in Fig. 6. While outside the scope of this study, membrane separation could also considerably reduce energy input for downstream upgrading of crude RCF oil in isolating the monomer fraction. A distillation modeled in Aspen Plus by Koelewijn *et al.*^74^ to isolate individual lignin monomers (*i.e.*, 4-propylsyringol from 4-propylguaiacol) required a 57-stage vacuum distillation column with a reflux ratio of 10 and estimated heat duty of 2.3 GJ per ton feed. Overall, further research in this area could be highly impactful for the sustainability of RCF-based biorefineries.

Sensitivity analysis of delignification revealed a trade-off between harsh conditions that achieve complete lignin extraction and the strong influence of reactor sizing on capital expenses and subsequent lignin oil cost results. This necessitates a shift from the simple goal of maximizing delignification, a metric generally used to compare processes in the literature, to balance the pressure and residence times that achieve higher lignin extraction and associated implications on RCF reactor costs. Thus, an ideal process could maximize the rate of lignin extraction to achieve high depolymerization at low residence times while simultaneously minimizing the RCF operating pressure. Note that this analysis assumed the polysaccharide pulp could be directly sent to enzymatic hydrolysis regardless of the extent of delignification, but further studies are necessary as enzymatic hydrolysis yields may be a function of delignification extent. Furthermore, this analysis focused on lignin fraction yield, but a similar sensitivity analysis on the extent of depolymerization as a function of reaction conditions would be required if the monomer fraction was the desired product stream. Finally, as this analysis was performed on poplar, further studies would be necessary with other feedstocks to determine the sensitivity of delignification to reaction conditions.

The low sensitivity of catalyst cost and catalyst lifetime on economic or environmental factors highlights the high relative costs of capital equipment and solvent recovery. For example, the cost of replacing 15% Ni/C catalyst at less than $1 MM per year is relatively low compared to natural gas ($55 MM per year), make-up methanol ($12 MM per year), or hydrogen ($5.5 MM per year) in the methanol case. As the operating and capital costs are reduced through the developments recommended in the previous paragraphs, it is expected that catalyst costs will become a more critical factor. Furthermore, the level of catalyst stability used in this analysis has not been achieved in the literature. Lan *et al.* showed a 50% reduction in monomer yields after flowing 1.2 g acetal-stabilized lignin over 0.125 g 5 wt% Ni/C catalyst, which would equate to approximately 33.7 kg biomass processed per kg 5 wt% Ni/C catalyst.^75^ Anderson *et al.* demonstrated a 10% decrease in monomer yields after processing four 1 g biomass beds over 0.15 g 15 wt% Ni/C, or 26.7 kg biomass per kg 15 wt% Ni/C, identifying sintering, leaching, and poisoning as the modes of deactivation.^29^ In this study, yearly replacement corresponds to 26 268 kg dry biomass per kg catalyst, and the sensitivity case of monthly replacement corresponds to 2189 kg biomass per kg catalyst. Monthly catalyst replacement could be seen as a medium-term objective to achieve catalyst costs that have minimal impact on process economics and limit excessive reactor down-time for catalyst replacement. We also recommend catalyst development for improved selectivity and operating conditions. Designing a catalyst to eliminate solvent losses due to alcohol reforming could reduce operating costs in the ethylene glycol case by $31 MM per year. Catalysts that can achieve complete lignin depolymerization at milder conditions, such as those used in the hydrogen-free case (which achieved the lowest MSP-lignin fraction of $1.34 per kg but highest MSP-monomer fraction of $7.58 per kg), could increase yields of monomeric products while maintaining lower capital and operating costs.

Polysaccharide retention during the RCF step had a surprisingly low impact in the sensitivity analysis of the methanol case in Fig. 3, and ethanol yields were not a significant cost driver in comparing processes in Fig. 5. This highlights an essential outcome of the models: when solving for RCF oil price in the overall integrated biorefinery models, crude RCF oil sales ($209 MM per year in the methanol case) account for more than twice the yearly revenue afforded by ethanol sales ($89 MM per year in the methanol case). Therefore, at this high valuation of RCF oil, it may be advantageous economically to improve lignin yields or reduce capital and operating costs even at the expense of carbohydrate retention. However, this conclusion depends on the availability of markets for RCF oil sales at this price point. As developments are made to reduce capital and operating costs, reduced lignin oil selling prices will increase the sensitivity of process economics to increases in ethanol production.

**Fig. 8:**
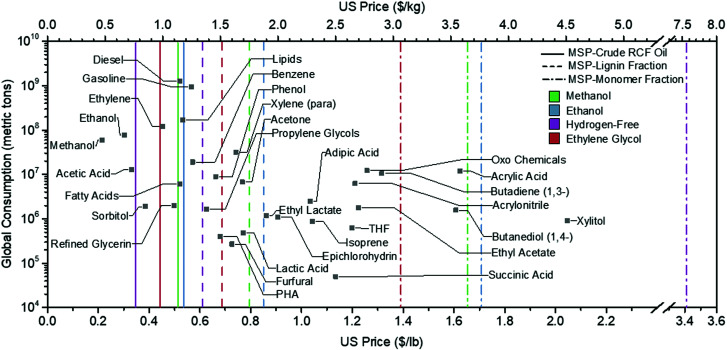
Minimum RCF oil selling prices with the U.S. price and global consumption of various chemicals for reference. The U.S. Price and Global Consumption data were sourced from a 2016 report by Biddy *et al.* and are 3–5 year averages.^76^ Tabulated data are summarized in Table S34 (ESI†).

To assess the feasibility of the MSPs calculated in this study, we plotted MSP-crude RCF oil, MSP-lignin fraction, and MSP-monomer fraction for each process configuration along with the price and global consumption of a number of chemicals (Fig. 8). The U.S. price and global consumption data were sourced from a 2016 report by Biddy *et al.* and show 3–5 year averages from the years 2010–2015.^76^ To utilize RCF oil in high-volume aromatic markets, such as phenol, benzene, and xylene, cost reductions would be necessary as well as downstream processing that enables the utilization of lignin oligomers in addition to monomers. To maximize the possibility for economic viability, we recommend developing processes that upgrade the entirety of crude RCF oil (including extracted carbohydrates, lignin monomers, and lignin oligomers). With a combination of cost reductions and efficient processes for increasing energy density (*e.g.*, through deoxygenation), crude RCF oil could be converted into diesel and gasoline.^36,77^ However, it should be noted that RCF oil contains ∼35 wt% oxygen, so deoxygenation to produce a hydrocarbon fuel would increase the price as normalized by oil weight.^36^ Some other proposed outlets for RCF oil were not included in the above-cited report. Additional market prices for vanillin, battery grade graphite, and BPA were $19.33 per kg, $26.46 per kg,^78^ and $1.54 per kg respectively. This reveals that monomeric products could be sold in high-value, low-volume markets of specialty chemicals such as vanillin, but reinforces that high-volume markets require the utilization of whole RCF oil feedstock and not just the monomer fraction in achieving profitability. For instance, Liao *et al.* showed that the monomer fraction could target phenol as an output, but only if the oligomer fraction was also utilized as printer ink.^41^ Furthermore, depending on the desired product stream, the RCF conditions and the choice of feedstock could tune the RCF oil composition (*e.g.* relative monomer *vs.* dimer content or distribution of specific monomers).^34^ In this analysis, we chose to remain agnostic to the downstream valorization strategies, intending that others can use this study to select a product portfolio rationally, and then optimize conditions accordingly.

A direct comparison of the LCA results presented here with other studies is not possible, as the previously reported applications of LCA to an RCF biorefinery are for different feedstocks and a different suite of co-products.^41,79^ The scope of the LCA was farm to biorefinery gate and did not include downstream processing or the use phase for the lignin fraction or ethanol. These additional processing steps and the use phase will increase the total GHG emissions generated without offering additional opportunities for carbon uptake. We also did not consider a specific biorefinery location in this work. Kumaniaev *et al.* found that locating the biorefinery near an existing pulp mill had a positive impact on LCA by integrating waste biomass streams and waste heat.^57^ Additional feedstock modeling is required to assess carbon uptake more precisely, accounting for biomass carbon uptake and location-specific carbon sequestration and releases from the soil and underground biomass (root systems). A follow-on analysis is planned for the methanol case process, in which a spatially explicit ecosystem model will be used to perform an entire carbon balance around the biorefinery system and the impacts from the downstream conversion of the RCF oil lignin fraction will be explored. This carbon balance analysis will provide a more accurate accounting of RCF-associated carbon sinks and sources. This analysis will also increase the lignin fraction CED calculation accuracy, as the farming process inputs (and resulting feedstock yield), transportation distances, and feedstock storage implications will be modeled in greater detail.

As currently estimated based on the design cases, the air emissions from utilizing lignin for RCF oil production are lower than those from the process utilizing lignin as boiler fuel for renewable diesel production documented in Davis *et al.* 2013,^1^ for which estimated emissions are shown in Eberle *et al.*^80^ The Davis *et al.* report used corn stover rather than poplar with a similar 2000 dry metric ton per day feedstock rate and burned the lignin extracted from dilute acid pretreatment. Burning lignin in the boiler for meeting the process heat and electricity demand resulted in CO, PM, NOx, and VOC emission rate of 198, 2.41, 42.6, and 7.32 g per GJ, respectively, *versus* 35.3, 0.0086, 26.7, and 2.15 g per GJ shown here from using natural gas in the methanol case. Although we use the best available emissions factors to quantify the emissions of criteria and HAPs for the design cases, these estimates should be considered preliminary, and further refinements would be needed once the information on process specifics (*e.g.*, the vapor pressure of RCF oil, HAP speciation of products) are made available from experiments and actual emission tests. It is also worth noting that potentially applicable federal (and state and local) regulations may require the adoption and installation of emission controls or work practice standards, which could also incur additional capital and operating costs, therefore affecting the MSP of the product.

## Materials and methods

### Property methods and property estimation

Given the non-ideality of the components used in the simulation, the NRTL-RK property method was chosen for all RCF area unit operations. The referenced Humbird *et al.* cellulosic ethanol model treats lignin as a waste product where its value is predominantly based on the process heat it produces *via* its combustion and thus assumed to be adequately represented as vanillin in process simulations. Given the complexity of lignin and its derivatives evolved through RCF chemistry, additional compounds were added to the present simulation to improve fidelity. Six monomers, two dimers, and two oligomers divided equally between S- and G-lignin constituents were incorporated into the model with the complete list of compounds shown in Fig. S8 (ESI†).

Except for 4-propylguaicol, found natively in the Aspen Plus databanks, all lignin derivative pure component thermodynamic and physical properties were estimated using the National Institute of Science and Technology ThermoDataEngine (TDE) capabilities built into the Aspen Plus software package.^81^ Binary interaction parameters were estimated using UNIFAC and fit to the NRTL-RK property method.

### Process economics

Economic assumptions were updated to be consistent with other recent TEA models, including cost year basis (2016), tax rate (21%), onstream time (90%), and plant startup time (0.5 years).^3^ For each process simulation, material, and energy flows calculated by the Aspen Plus process model were imported into an Excel spreadsheet, accounting for capital and operational costs. Given two products in the biorefinery, ethanol and RCF oil, the selling price of ethanol was fixed to be $1.65 per gal (equivalent to $2.50 per gallon of gasoline-equivalent on an energy basis), and the minimum RCF oil selling price was determined using a discounted cash flow rate of return analysis to achieve a net present value of zero assuming an after-tax rate of return of 10% over the 30 year lifespan of the biorefinery. Minimum lignin oil selling price and minimum lignin monomer selling price were determined by dividing the minimum RCF oil selling price by the mass fraction of total lignin components (monomers, dimers, and oligomers) or only the monomers present in the oil, respectively. These prices do not include any additional separations required to isolate the fractions from the crude RCF oil.

### Capital costs

All non-RCF area capital equipment base costs, scaling exponents, and installation factors were identical to that of the Humbird *et al.* report adjusted to a 2016 cost index.^46^ In the RCF area, pumps, compressors, distillation columns, and flash drums were costed in Aspen Capital Cost Evaluator (ACCE) V10 using flowrates and operating conditions imported from the results of the Aspen Plus simulation with default costing assumptions and a 2016 cost year. Costs and operational metrics for centrifuges and PSA hydrogen recovery units were estimated from other NREL reports.^60,82^

While software and empirical correlations exist for sizing and costing standard equipment such as pumps, compressors, distillation columns, and common reactor types, novel reactor types typically lack these costing tools. To develop capital cost estimates for the RCF reactor, a quote for a pulping reactor initially prepared by the Harris Group Inc. for NREL, referenced in a previous report^1^ was used as a basis. The quoted reactor was originally designed for lignin solubilization from whole biomass using similar feed flow rate, solids loading, and reactor temperature to those found in our proposed RCF reactor, although design pressure was significantly less at only 6 bar. To estimate reactor cost as a function of pressure, a series of vertical pressure vessels of similar size and operating conditions to the RCF reactor were costed in ACCE V10. Linear regression was used to develop a pressure scaling factor applied to the base cost, normalizing costs to the 6 bar basis up to 60 bar. The installation factor was assumed to be 1.7, and the scaling exponent was assumed to be 0.60, both values provided by Harris Group Inc.

### Operating costs

Variable operating costs for raw materials, wastes, utilities, and process byproducts were determined from the Aspen Plus process simulation results. While the economic analysis maintains a majority of cost assumptions used by Humbird *et al.*^46^ several additions to materials and catalysts were incorporated into the model to account for RCF area materials and natural gas imports to the boiler for process heat. Additional material costing assumptions are summarized in Table 4.

**Table 4:** Operating costs. Summary of variable operating cost additions on top of those found already present in the Humbird *et al.* model^46^

Component	Cost (2016$)	Source
Poplar feedstock	$80 per dry U.S. ton ($88.18 per dry metric ton)	Billion-Ton Study,^85^ Greenwood resources,^86^ Happs *et al.*^62^
Methanol	$0.27455 per kg	Industry database
Ethylene glycol	$0.8192 per kg	Industry database
Hydrogen	$1.6106 per kg	Davis *et al.* 2018 Design Report^3^
Natural gas	$0.2612 per kg ($5 per MMBtu)	Davis *et al.* 2018 Design Report^3^
15% Ni/C catalyst	$37.48 per kg (net)	CatCost estimate: $35.91 per kg purchase cost with $1.57 per kg disposal cost^84^
5% Pd/C catalyst	$224.75 per kg (net)	CatCost estimate: $1539.40 per kg purchase cost with $1314.65 per kg spent catalyst value^84^

Catalyst cost estimates were generated using the CatCost tool^83,84^ assuming 14 ton order sizes (twice per year) and a 2016 cost basis. Estimated delivered cost for poplar feedstock varies depending on total demand, harvest interval, and biorefinery location.^85,86^ We assume here a delivered cost of $80 per dry U.S. ton ($88.18 per dry metric ton), similar to costs for woody feedstocks assumed in other reports.^60–62^

### Life cycle assessment

**Fig. 9:**
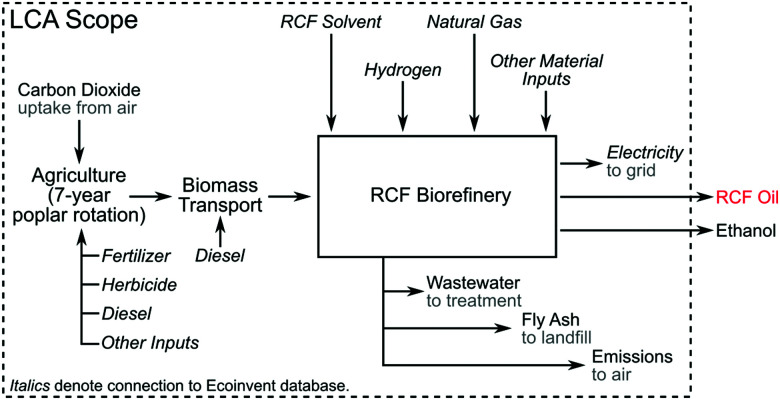
LCA system boundary diagram, with connections to the background LCI database indicated with italics.

Life cycle models of the methanol, ethanol, hydrogen-free, ethylene glycol, and membrane RCF cases were developed and used as the basis for a univariate sensitivity analysis and impact breakdown by process input and by process area. A system boundary diagram of the LCA is given in Fig. 9. Membrane production and transportation was excluded from the system boundary for the EG-membrane RCF case, due to a lack of reliable data for those processes. Process-level material and energy use and direct CO_2_ emissions were obtained from the Aspen Plus simulations used as the basis for the TEA.^55^ The LCA modeling software used was SimaPro version 9.0, with the DATASMART life cycle inventory database as the primary source of background process data.^54,87^

Additional background data, including the poplar farming process, was obtained from Dunn *et al.*^55^ The poplar was assumed to be purpose-grown as RCF biorefinery feedstock, and the impacts from direct and indirect land-use change were not quantified. Quantifying land-use change impacts requires modeling a specific spatial location for feedstock agriculture, and in this study, a representative farming model was used. In future work, the impacts of direct land-use change will be quantified with a spatially explicit ecosystem model.

### Air pollutant emissions

The air pollutants that are likely to be emitted from the process are identified based on the process model and discussion with process design engineers. For this analysis, only air emissions regulated by the EPA under the Clean Air Act (CAA) were considered. Emissions are estimated using material balance for process design, EPA's Compilation of Air Pollution Emission Factors Report (AP-42), EPA guidance documents (*e.g.*, for equipment leak estimation), and predictive models (*e.g.*, TANKS). The emissions reflect the greatest amount of air pollutants that a plant could emit under its physical and operational design, but without considering limits, which applicable federal regulations could require. Refer to ESI† for detailed methodology, control technologies considered, and emission factors utilized for determining emissions from each unit operation of the process.

## Conflicts of interest

None to declare.

## Supplementary Material

EE-014-D1EE01642C-s001
