# Supplementary material for: Techno-economic analysis and life cycle assessment of a biorefinery utilizing reductive catalytic fractionation
Source: Energy Environ Sci. 2021 Jul 8;14(8):4147–68. doi: 10.1039/d1ee01642c (PMC9562980; doi:10.1039/d1ee01642c)
Supplement: EE-014-D1EE01642C-s001 [file EE-014-D1EE01642C-s001.pdf]

Electronic Supplementary Material (ESI) for Energy & Environmental Science.
This journal is © The Royal Society of Chemistry 2021

Supporting Information

Techno-economic analysis and life cycle assessment of a biorefinery utilizing reductive catalytic fractionation

Andrew W. Bartling,[1,2] Michael L. Stone,[3] Rebecca J. Hanes,[2,4] Arpit Bhatt[4], Yimin Zhang[4], Mary J. Biddy,[1,2] Ryan Davis,[1] Jacob S. Kruger,[5] Nicholas E. Thornburg,[1] Jeremy S. Luterbacher,[6] Roberto Rinaldi,[7] Joseph S. M. Samec,[8] Bert F. Sels,[9] Yuriy Román-Leshkov,[3,*] and Gregg T. Beckham[2,5,*]

1. Catalytic Carbon Transformation and Scale-Up Center, National Renewable Energy Laboratory, Golden CO, 80401 USA
2. Center for Bioenergy Innovation, Oak Ridge TN 37830 USA
3. Department of Chemical Engineering, Massachusetts Institute of Technology, Cambridge MA 02139 USA
4. Strategic Energy Analysis Center, National Renewable Energy Laboratory, Golden CO, 80401 USA
5. Renewable Resources and Enabling Sciences Center, National Renewable Energy Laboratory, Golden CO 80401 USA
6. Laboratory of Sustainable and Catalytic Processing, Institute of Chemical Sciences and Engineering École Polytechnique Fédérale de Lausanne (EPFL), CH-1015 Lausanne, Switzerland
7. Department of Chemical Engineering, Imperial College London, South Kensington Campus, London SW7 2AZ, UK
8. Department of Organic Chemistry, Stockholm University, SE-106 91 Stockholm, Sweden
9. Center for Sustainable Catalysis and Engineering, KU Leuven, Celestijnenlaan 200F, 3001 Leuven, Belgium

* Email: gregg.beckham@nrel.gov, yroman@mit.edu

lignin valorization | biomass fractionation | biofuels | biochemicals

List of Abbreviations:

- ACCE – Aspen Capital Cost Estimator
- CAA – Clean Air Act
- CED – Cumulative energy demand
- CHP – Combined heat and power
- CO – Carbon monoxide
- CUB – Catalytic upstream biorefining
- EG-Membrane – Ethylene glycol membrane
- EPA – Environmental Protection Agency
- GHG – Greenhouse Gas
- GWP – Global warming potential
- HAP – Hazardous Air Pollutants
- LCA – Life cycle assessment
- LHV – Lower heating value
- MM – Million
- MSP – Minimum selling price
- NOx – Nitrogen oxides
- NREL – National Renewable Energy Laboratory
- NRTL-RK – Nonrandom Two-Liquid-Redlich-Kwong
- OSN – Organic solvent nanofiltration
- PM – Particulate matter
- PSA – Pressure swing adsorption
- RCF – Reductive catalytic fractionation
- SOx – Sulfur oxides
- STP – Standard temperature and pressure (0 ºC, 1 atm)
- TDE – ThermoDataEngine
- TEA – Techno-economic analysis
- TIC – Total installed capital
- TPY – Tons per year
- UNIFAC - Universal Quasichemical Functional-group Activity Coefficients
- USD – United States Dollar (2016$ basis)
- VOC – Volatile organic compound
- WWT – Wastewater treatment

Table S1. Feedstock Composition.

Component	Weight % (Dry Basis)
Cellulose	46.4%
Xylan	13.4%
Arabinan	0.2%
Mannan	3.7%
Galactan	1.4%
Sucrose	0.1%
Lignin	28.5%
Acetate	3.5%
Extractives	1.6%
Ash	1.1%
Moisture Content	20%

Table S2. Flowrates (kg/hr) of Major Process Components Present in Aspen Plus Simulation. Refer to **Fig. S1** for overview of process configuration and stream names. Note that due to minor purge streams and splits not captured in the process flow diagram (**Fig. S1**), mass balances around individual unit operations may not close to 100%, although flows in and out of the system boundary maintain fidelity.

**Stream ID**	**A**	**B**	**C**	**D**	**E**	**F**	**G**
H2O	0	0	20833.3	1345.1	0	176.5	93983.4
Ethanol	0	0	0	0	0	0	0
Glucose	0	0	0	0	0	0	1561.4
Galactose	0	0	0	0	0	0	235.5
Mannose	0	0	0	0	0	0	622.4
Xylose	0	0	0	0	0	0	323.0
Arabinose	0	0	0	0	0	0	41.3
Cellobiose	0	0	0	0	0	0	0
Sucrose	0	0	83.3	0	0	0	100.9
Reformed/Decomposed Sugars	0	0	0	0	0	0	5829.3
Extractives	0	0	1333.3	0	0	0	1613.0
Lactic Acid	0	0	0	0	0	0	0
Xylitol	0	0	0	0	0	0	0
Glycerol	0	0	0	0	0	0	0
Succinic Acid	0	0	0	0	0	0	0
NH3	0	0	0	0	0	0	0
Diammonium Phosphate	0	0	0	0	0	0	0
Ethanol Denaturant	0	0	0	0	0	0	0
CO	0	0	0	1173.1	0	1172.3	384.2
CO2	0	0	0	1877.2	0	1841.8	410.8
Cellulose	0	0	38706.0	0	0	0	34851.1
Galactan	0	0	1167.9	0	0	0	584.1
Mannan	0	0	3086.5	0	0	0	1543.6
Xylan	0	0	11178.0	0	0	0	10400.4
Arabinan	0	0	166.8	0	0	0	66.7
Solid Lignin	0	0	23774.2	0	0	0	7133.3
Acetate	0	0	2919.6	0	0	0	2921.1
Protein	0	0	0	0	0	0	0
Ash	0	0	917.6	0	0	0	918.1
Enzymes	0	0	0	0	0	0	0
Denatured Enzyme	0	0	0	0	0	0	0
z. mobilis	0	0	0	0	0	0	0
t. reesei	0	0	0	0	0	0	0
Hydrogen Gas	432.8	0	0	4780.8	4063.7	716.6	0
Methanol	0	5593.6	0	15921.7	0.0	2235.4	517800.1
Total Solubilized Lignin	0	0	0	0.1	0	0.2	19736.8
Solubilized Lignin Monomers	0	0	0	0.1	0	0.2	9681.7
Solubilized Lignin Dimers	0	0	0	0	0	0	5026.7
Solubilized Lignin Oligomers	0	0	0	0	0	0	5028.4

**Stream ID**	**H**	**I**	**J**	**K**	**L**	**M**	**N**
H2O	74101.5	19881.8	71693.1	2408.4	71711.0	20080.9	112.4
Ethanol	0	0	0	0	0	0	0
Glucose	1231.1	330.3	0	1231.1	0.4	0.7	1230.4
Galactose	185.7	49.8	0	185.7	0.1	0.1	185.6
Mannose	490.7	131.7	0	490.7	0.2	0.3	490.5
Xylose	254.7	68.3	0	254.7	0.3	0.3	254.4
Arabinose	32.6	8.7	0	32.6	0	0	32.5
Cellobiose	0	0	0	0	0	0	0
Sucrose	79.6	21.3	0	79.6	0.1	0.1	79.5
Reformed/Decomposed Sugars	4596.1	1233.2	0	4596.1	2.6	3.1	4593.0
Extractives	1271.8	341.2	0	1271.8	0.5	0.7	1271.0
Lactic Acid	0	0	0	0	0	0	0
Xylitol	0	0	0	0	0	0	0
Glycerol	0	0	0	0	0	0	0
Succinic Acid	0	0	0	0	0	0	0
NH3	0	0	0	0	0	0	0
Diammonium Phosphate	0	0	0	0	0	0	0
Ethanol Denaturant	0	0	0	0	0	0	0
CO	302.9	81.3	302.9	0	389.3	0	0
CO2	323.9	86.9	323.9	0	458.4	0	0
Cellulose	17.4	34833.7	0	17.4	0	0	17.4
Galactan	0.3	583.8	0	0.3	0	0	0.3
Mannan	0.8	1542.9	0	0.8	0	0	0.8
Xylan	5.2	10395.2	0	5.2	0	0	5.2
Arabinan	0	66.7	0	0.03	0	0	0.03
Solid Lignin	3.6	7129.8	0	3.6	0	0	3.6
Acetate	1.5	2919.6	0	1.5	0	0	1.5
Protein	0	0	0	0	0	0	0
Ash	0.5	917.6	0.0	0.5	0	0	0.5
Enzymes	0	0	0	0	0	0	0
Denatured Enzyme	0	0	0	0	0	0	0
z. mobilis	0	0	0	0	0	0	0
t. reesei	0	0	0	0	0	0	0
Hydrogen Gas	0	0	0	0	0.6	0	0
Methanol	408261.4	109538.7	408057.3	204.1	539176.8	593.5	18.7
Total Solubilized Lignin	15561.5	4175.2	16.8	15544.7	96.9	275.4	15286.2
Solubilized Lignin Monomers	7633.6	2048.1	16.8	7616.8	96.9	275.4	7358.2
Solubilized Lignin Dimers	3963.3	1063.4	0	3963.3	0	0	3963.3
Solubilized Lignin Oligomers	3964.6	1063.7	0	3964.6	0	0	3964.6

Stream ID	O	P	Q	R	S	T	U
H2O	91592.9	74946.2	16646.0	11.8	234578.8	262028.7	104.2
Ethanol	0	0	0	0	21.1	20823.0	20666.2
Glucose	330.7	270.6	60.1	59.7	34875.9	612.3	0
Galactose	49.9	40.8	9.1	9.0	9.0	8.7	0
Mannose	131.8	107.9	24.0	23.8	23.8	23.1	0
Xylose	68.6	56.2	12.5	12.2	10047.9	647.6	0
Arabinose	8.8	7.2	1.6	1.5	1.5	0.2	0
Cellobiose	0	0	0	0	441.0	441.0	0
Sucrose	21.5	17.6	3.9	3.8	3.8	3.8	0
Reformed/Decomposed Sugars	1235.8	1011.2	224.6	222.0	1614.6	1614.6	0
Extractives	341.8	279.7	62.1	61.6	61.6	61.6	0
Lactic Acid	0	0	0	0	54.9	1469.5	0
Xylitol	0	0	0	0	0	460.4	0
Glycerol	0	0	0	0	0	169.8	0
Succinic Acid	0	0	0	0	0	384.8	0
NH3	0	0	0	0	0.3	0.3	0.3
Diammonium Phosphate	0	0	0	0	0	82.3	0
Ethanol Denaturant	0	0	0	0	0	0	444.2
CO	470.6	385.1	85.5	0	0	0	0
CO2	545.3	446.2	99.1	0	1.1	403.6	20.3
Cellulose	34833.7	17.4	34816.3	34816.3	1671.2	1671.2	0
Galactan	583.8	0.3	583.5	583.5	583.5	583.5	0
Mannan	1542.9	0.8	1542.1	1542.1	1542.1	1542.1	0
Xylan	10395.2	5.2	10390.0	10390.0	1558.5	1558.5	0
Arabinan	66.7	0.03	66.7	66.7	66.7	66.7	0
Solid Lignin	7129.8	3.6	7126.2	7126.2	7126.2	7126.2	0
Acetate	2919.6	1.5	2918.2	2918.2	2918.2	2918.2	0
Protein	0	0	0	0	54.8	203.9	0
Ash	917.6	0.5	917.1	917.1	917.1	917.1	0
Enzymes	0	0	0	0	693.7	693.7	0
Denatured Enzyme	0	0	0	0	77.1	77.1	0
z. mobilis	0	0	0	0	0	787.8	0
t. reesei	0	0	0	0	143.6	143.6	0
Hydrogen Gas	0.6	0.5	0.1	0	0	0	0
Methanol	648715.6	530813.6	117896.9	55.6	59.0	59.1	43.0
Total Solubilized Lignin	4272.1	3495.7	776.4	679.4	679.5	679.5	0
Solubilized Lignin Monomers	2145.0	1755.2	389.9	292.8	292.9	292.9	0
Solubilized Lignin Dimers	1063.4	870.1	193.3	193.3	193.3	193.3	0
Solubilized Lignin Oligomers	1063.7	870.4	193.3	193.3	193.3	193.3	0

Table S3. Allocation factors for the five RCF cases. All economic allocation factors were calculated using sale prices of $1.74/kg for lignin fraction and $1.65/gal for ethanol.

RCF Case	Co-Product	Mass Allocation Factor	Economic Allocation Factor
**Methanol**	Lignin Fraction	0.424	0.699
	Ethanol	0.576	0.301
**Ethanol**	Lignin Fraction	0.439	0.711
	Ethanol	0.561	0.289
**Hydrogen-Free**	Lignin Fraction	0.489	0.751
	Ethanol	0.511	0.249
**Ethylene Glycol**	Lignin Fraction	0.435	0.708
	Ethanol	0.565	0.292
**EG-Membrane**	Lignin Fraction	0.433	0.706
	Ethanol	0.567	0.294

Table S4. Methanol Case Summary.

Methanol Case Summary

(All Values in 2016$)

MSP-Crude RCF Oil	**$1.13 /kg**
MSP-Lignin Fraction	**$1.74 /kg**
MSP-Monomer Fraction	**$3.63 /kg**
Ethanol Selling Price	**$1.65 /gal**
Crude RCF Oil Production	185.9 MMkg/yr
Crude RCF Oil Yield	283.0 kg/dry metric ton
Ethanol Production	206.5 MML/yr (Ethanol at 68 °F)
Ethanol Yield	314.3 L / dry metric ton feedstock
Feedstock Cost	$88.18 /dry metric ton
Internal Rate of Return (After-Tax)	10%
Equity Percent of Total Investment	40%

Capital Costs	
RCF Area (Reactor Only Costs)	$290,362,170
RCF Area (Non-Reactor Costs)	$33,147,597
Saccharification & Fermentation	$23,700,000
On-site Enzyme Production	$23,200,000
Distillation and Solids Recovery	$21,000,000
Wastewater Treatment	$43,500,000
Storage	$4,400,000
Boiler/Turbogenerator	$114,400,000
Utilities	$10,000,000
Total Installed Equipment Cost	$563,709,766
Added Direct + Indirect Costs	$500,090,234
(% of TCI)	47%
Total Capital Investment (TCI)	$1,063,800,000
Lignin Yield (kg/dry metric ton)	183.7
(wt.% biomass lignin)	64.3%
Monomer Yield (kg/dry metric ton)	88.4
(wt.% biomass lignin)	31.0%
Maximum Ethanol Yields (100% of Theoretical)	
Ethanol Production (MML/yr)	310.2
Theoretical Yield (L/dry metric ton)	472.2
Current Yield (Actual/Theoretical)	66.6%
RCF Area Heat Demand (MJ/s)	295.2
(% biomass energy LHV)	72.8%
Natural Gas Imports (kg/kg crude RCF oil)	1.14
Excess Electricity (kWh/kg crude RCF oil)	1.5
Plant Water Usage (L/kg crude RCF oil)	7.8

RCF Oil Composition (wt.%)	
Total Lignin	64.9%
Monomers	31.2%
Dimers	16.8%
Oligomers	16.8%
Carbohydrate Derivatives	29.1%
Extractives	5.4%
Residual Solvent/Water	0.6%

Manufacturing Costs (cents/kg crude RCF oil)	
Feedstock	31.3
Methanol	6.5
Hydrogen	3.0
Glucose (enzyme production)	10.5
Catalysts	0.5
Other Raw Materials	3.0
Natural Gas	29.7
Waste Disposal	0.2
Net Electricity	-8.8
Ethanol	-48.5
Fixed Costs	13.1
Capital Depreciation	18.2
Average Income Tax	3.7
Average Return on Investment	51.1

Manufacturing Costs ($/yr)	
Feedstock	$57,900,000
Methanol	$12,100,000
Hydrogen	$5,500,000
Glucose (enzyme production)	$19,400,000
Catalysts	$900,000
Other Raw Materials	$5,500,000
Natural Gas	$55,000,000
Waste Disposal	$400,000
Net Electricity	-$16,400,000
Ethanol	-$89,700,000
Fixed Costs	$24,300,000
Capital Depreciation	$33,700,000
Average Income Tax	$6,800,000
Average Return on Investment	$94,500,000

RCF Specific Operating Conditions	
RCF Reactor Temperature (°C)	200
RCF Reactor Pressure (bar)	60
RCF Residence Time (h)	3
Reactor Solvent Loading (L/dry kg biomass)	9
Biomass Delignification	70%
RCF Reactor Total Solids	12.7%
Net RCF Oil Recovery	91.8%

Table S5. Breakdown of contributions to methanol case lignin fraction GWP and CED based on economic allocation.

Process Area	GWP-Lignin Fraction (kg CO_2eq/kg)	CED-Lignin Fraction (MJ/kg)
Delivered Feedstock	-6.80	70.22
RCF Area	6.36	61.83
Hydrolysis & Fermentation Areas	0.02	0.32
Ethanol Recovery	1.60	5.91
On-Site Enzyme Production	0.26	4.19
Utilities	6.08E-04	0.01
Wastewater Treatment (WWT) Area	2.71E-08	5.24E-07
Electricity Offset	-1.30	-21.29

Table S6. Tabulated data from Main Text Figure 2a. Costs are in USD/kg lignin fraction.

Process Area	Capital Recovery Charge	Raw Materials, Catalyst, & Waste	Process Electricity	Grid Electricity	Ethanol	Total Plant Electricity	Fixed Costs	Area Totals
Feedstock & Handling	0	0.480	0	0	0	0	0	0.480
RCF	0.642	0.154	0.016	0	0	0	0.116	0.927
Enzymatic Hydrolysis & Fermentation	0.047	0.015	0.007	0	0	0	0.008	0.078
Enzyme Production	0.046	0.185	0.027	0	0	0	0.008	0.265
Recovery	0.042	0	0.006	0	0	0	0.008	0.055
Wastewater Treatment	0.086	0	0.021	0	0	0	0.016	0.123
Storage	0.009	0	0	0	0	0	0.002	0.010
Boiler/Turbogenerator	0.227	0.459	0.010	-0.136	0	-0.114	0.041	0.488
Utilities	0	0	0	0	0.000	0	0.004	0.057
Ethanol Sales	0	0	0	0	-0.744	0	0	-0.744
Category Totals	1.119	1.298	0.114	-0.136	-0.744	-0.114	0.201	1.739

Table S7. Tabulated data from Main Text Figure 2b-c.

Process Area	GWP-Lignin Fraction (kg CO_2eq/kg)	CED-Lignin Fraction (MJ/kg)
Delivered Feedstock	-4.13	42.93
RCF Area	3.86	37.8
Hydrolysis & Fermentation Areas	0.01	0.20
Ethanol Recovery	0.97	3.61
On-Site Enzyme Production	0.16	2.56
Utilities	3.69E-04	0.01
Wastewater Treatment (WWT) Area	1.64E-08	3.21E-07
Electricity Offset	-0.79	-13.01

Table S8. Rationale of chosen minima/maxima for single-point sensitivity analysis

Sensitivity Analysis Parameter (low cost:base:high cost)	Rationale of Minima/Maxima
RCF Area Capital Cost (%) 50:100:150	±50% chosen due to uncertainty in capital cost estimation of RCF reactor.
Delignification (%) 80:70:60	±10% chosen to evaluate variability of delignification in RCF or changes/modifications to feedstock based on existing literature studies.[1,2]
Reactor Residence Time (h) 1:3:5	±2 hour to reflect potential for residence time improvements with improved rate of lignin solvolysis or evaluate tradeoffs of longer residence times. Studies have shown minimal additional RCF oil yield above 5-hour reaction time.[3]
Ethanol Selling Price ($/gal) 1.97:1.65:1.32	Evaluates lignin co-product sensitivity to potential market fluctuations in fuel selling price. Equivalent to ±$0.50/gallon gasoline equivalent (LHV)
Feedstock Cost ($/dry US ton) 60:80:100	Reflects uncertainty and variability in delivered poplar feedstock cost based on literature review.[4]
Reactor Pressure (bar) 50:60:70	±10 bar due to approach of limit of saturation pressure to maintain sufficient methanol solvent in liquid phase at reactor temperature.
Solvent Loading (L/dry kg biomass) 8:9:10	Additional sensitivity and more detail of solvent loading is given in main text.
Catalyst Lifetime (months) 36:12:1	Approximates the potential and uncertainty for catalyst lifetime variability as process scales and catalyst stability improves. Provides value proposition to improvements to catalyst lifetime.
Methanol Decomposition (%) 0:0.5:1	±0.5% chosen due to limited information of solvent mass balance in literature to potential upper and lower bounds based on catalyst selection and to give value proposition to catalyst improvements.[5]
Catalyst Cost ($/kg) 3.75:37.5:374.8	Order of magnitude chosen to reflect potential catalyst cost variation depending on catalyst selection.[6]
Cellulose Retention (%) 100:90:80	Reflects uncertainty and variability of cellulose retention using methanol-rich RCF solvents and provides value proposition to improving retention.[7,8]
Xylose Retention (%) 100:93:80	Reflects uncertainty and variability of xylose retention using methanol-rich RCF solvents and provides value proposition to improving retention.[7,8]

Table S9. Tabulated data from Main Text Figure 3.

Sensitivity Analysis Parameter (low cost:base:high cost)	Change to MSP-Lignin Fraction (USD/kg)		Change to GWP-Lignin Fraction (kg CO_2-eq/kg)		Change to CED-Lignin Fraction (MJ/kg)	
	Low Cost	High Cost	Low Cost	High Cost	Low Cost	High Cost
RCF Area Capital Cost (%) 50:100:150	-0.39	0.39	-	-	-	-
Delignification (%) 80:70:60	-0.21	0.29	-0.04	0.05	-2.69	3.80
Reactor Residence Time (h) 1:3:5	-0.13	0.20	-	-	-	-
Ethanol Selling Price ($/gal) 1.97:1.65:1.32	-0.15	0.15	-	-	-	-
Feedstock Cost ($/dry US ton) 60:80:100	-0.12	0.12	-	-	-	-
Reactor Pressure (bar) 50:60:70	-0.10	0.10	-0.07	0.08	-0.89	0.87
Solvent Loading (L/dry kg biomass) 8:9:10	-0.09	0.09	-0.25	0.27	-2.80	3.02
Catalyst Lifetime (months) 36:12:1	-0.01	0.09	0.00	0.00	0.00	0.07
Methanol Decomposition (%) 0:0.5:1	-0.05	0.03	-0.27	0.22	-3.74	3.05
Catalyst Cost ($/kg) 3.75:37.5:374.8	-0.01	0.07	-	-	-	-
Cellulose Retention (%) 100:90:80	-0.02	0.02	0.12	-0.12	-2.76	3.19
Xylose Retention (%) 100:93:80	-0.01	0.02	0.02	-0.02	-0.72	1.79

Table S10. Sensitivity results for change to MSP-Crude RCF Oil. Note that reductions in both cellulose retention and xylose retention lead to net reductions in MSP-Crude RCF Oil due to inflated crude RCF oil yields.

	Change to MSP-Crude RCF Oil (USD/kg)	
**Sensitivity Analysis Parameter (low cost:base:high cost)**	**Low Cost**	**High Cost**
RCF Area Capital Cost (%) 50:100:150	-0.25	0.25
Cellulose Retention (%) 80:90:100	-0.15	0.20
Reactor Residence Time (h) 1:3:5	-0.09	0.13
Delignification (%) 80:70:60	-0.09	0.11
Ethanol Selling Price ($/gal) 1.97:1.65:1.32	-0.10	0.10
Feedstock Cost ($/dry US ton) 60:80:100	-0.08	0.08
Reactor Pressure (bar) 50:60:70	-0.06	0.07
Solvent Loading (L/dry kg biomass) 8:9:10	-0.06	0.06
Xylose Retention (%) 80:93:100	-0.06	0.03
Catalyst Lifetime (months) 36:12:1	0.00	0.06
Methanol Decomposition (%) 0:0.5:1	-0.03	0.02
Catalyst Cost ($/kg) 3.75:37.5:374.8	0.00	0.05

Table S11. Sensitivity results for methanol case lignin fraction GWP and CED based on economic allocation.

	Change to GWP-Lignin Fraction (kg CO2-eq/kg)		Change to CED-Lignin Fraction(MJ/kg)	
**Sensitivity Analysis Parameter (low cost:base:high cost)**	**Low Cost**	**High Cost**	**Low Cost**	**High Cost**
Delignification (%) 80:70:60	-0.061	0.093	-9.37	11.63
Reactor Pressure (bar) 50:60:70	-0.107	0.137	-1.48	1.43
Solvent Loading (L/dry kg biomass) 8:9:10	-0.411	0.449	-4.62	4.78
Catalyst Lifetime (months) 36:12:1	-0.000	0.006	-0.01	0.11
Methanol Decomposition (%) 0:0.5:1	-0.438	0.362	-6.17	5.02
Cellulose Retention (%) 100:90:80	0.204	-0.191	-1.75	2.04
Xylose Retention (%) 100:93:80	0.025	-0.027	-0.71	1.93

Table S12. Tabulated data from Main Text Figure 4.

Total Solvent Loading (L/dry kg biomass)	MSP-Lignin Fraction (USD/kg)	MSP-Crude RCF Oil (USD/kg)	GWP-Lignin Fraction (kg CO_2-eq/kg)	CED-Lignin Fraction (MJ/kg)
4.0	1.41	0.90	-1.08	62.62
5.0	1.43	0.93	-0.89	63.81
6.0	1.49	0.97	-0.67	65.78
7.0	1.57	1.02	-0.42	68.54
8.0	1.65	1.07	-0.17	71.24
9.0	1.74	1.13	0.08	74.03
10.0	1.83	1.19	0.35	77.05

Table S13. Solvent Loading Sensitivity results for methanol case lignin fraction GWP and CED based on economic allocation.

Total Solvent Loading (L/dry kg biomass)	GWP-Lignin Fraction (kg CO_2-eq/kg)	CED-Lignin Fraction (MJ/kg)
4.0	-1.84	107.07
5.0	-1.50	107.23
6.0	-1.11	109.62
7.0	-0.69	113.53
8.0	-0.28	117.42
9.0	0.13	121.99
10.0	0.58	126.82

Table S14. Ethanol Case Summary.

Ethanol Case Summary

(All Values in 2016$)

MSP-Crude RCF Oil	**$1.18 /kg**
MSP-Lignin Fraction	**$1.88 /kg**
MSP-Monomer Fraction	**$3.76 /kg**
Ethanol Selling Price	**$1.65 /gal**
Crude RCF Oil Production	169.4 MMkg/yr
Crude RCF Oil Yield	257.9 kg/dry metric ton
Ethanol Production	172.9 MML/yr (Ethanol at 68 °F)
Ethanol Yield	263.2 L / dry metric ton feedstock
Feedstock Cost	$88.18 /dry metric ton
Internal Rate of Return (After-Tax)	10%
Equity Percent of Total Investment	40%

Capital Costs	
RCF Area (Reactor Only Costs)	$256,396,249
RCF Area (Non-Reactor Costs)	$31,116,741
Saccharification & Fermentation	$24,100,000
On-site Enzyme Production	$25,000,000
Distillation and Solids Recovery	$20,800,000
Wastewater Treatment	$45,400,000
Storage	$3,900,000
Boiler/Turbogenerator	$106,700,000
Utilities	$9,500,000
Total Installed Equipment Cost	$522,912,989
Added Direct + Indirect Costs	$462,287,011
(% of TCI)	47%
Total Capital Investment (TCI)	$985,200,000
Lignin Yield (kg/dry metric ton)	162.4
(wt.% biomass lignin)	56.8%
Monomer Yield (kg/dry metric ton)	81.0
(wt.% biomass lignin)	28.3%
Maximum Ethanol Yields (100% of Theoretical)	
Ethanol Production (MML/yr)	310.2
Theoretical Yield (L/dry metric ton)	472.2
Current Yield (Actual/Theoretical)	55.7%
RCF Area Heat Demand (MJ/s)	257.3
(% biomass energy LHV)	63.5%
Natural Gas Imports (kg/kg crude RCF oil)	1.08
Excess Electricity (kWh/kg crude RCF oil)	1.4
Plant Water Usage (L/kg crude RCF oil)	6.2

RCF Oil Composition (wt.%)	
Total Lignin	63.0%
Monomers	31.4%
Dimers	15.8%
Oligomers	15.8%
Carbohydrate Derivatives	30.7%
Extractives	5.9%
Residual Solvent/Water	0.5%

Manufacturing Costs (cents/kg crude RCF oil)	
Feedstock	34.2
Methanol	0.0
Hydrogen	1.9
Glucose (enzyme production)	12.1
Catalysts	3.3
Other Raw Materials	3.3
Natural Gas	28.3
Waste Disposal	0.2
Net Electricity	-8.0
Ethanol	-44.3
Fixed Costs	0.0
Capital Depreciation	18.4
Average Income Tax	3.9
Average Return on Investment	64.9

Manufacturing Costs ($/yr)	
Feedstock	$57,900,000
Methanol	$0
Hydrogen	$3,300,000
Glucose (enzyme production)	$20,500,000
Catalysts	$5,600,000
Other Raw Materials	$5,500,000
Natural Gas	$48,000,000
Waste Disposal	$400,000
Net Electricity	-$13,500,000
Ethanol	-$75,100,000
Fixed Costs	$22,700,000
Capital Depreciation	$31,200,000
Average Income Tax	$6,500,000
Average Return on Investment	$110,000,000

RCF Specific Operating Conditions	
RCF Reactor Temperature (°C)	200
RCF Reactor Pressure (bar)	50
RCF Residence Time (h)	3
Reactor Solvent Loading (L/dry kg biomass)	9
Biomass Delignification	60%
RCF Reactor Total Solids	12.8%
Net RCF Oil Recovery	90.6%

Table S15. Hydrogen-Free Case Summary.

Hydrogen-Free Case Summary

(All Values in 2016$)

MSP-Crude RCF Oil	**$0.76 /kg**
MSP-Lignin Fraction	**$1.34 /kg**
MSP-Monomer Fraction	**$7.58 /kg**
Ethanol Selling Price	**$1.65 /gal**
Crude RCF Oil Production	232.8 MMkg/yr
Crude RCF Oil Yield	354.3 kg/dry metric ton
Ethanol Production	174.4 MML/yr (Ethanol at 68 °F)
Ethanol Yield	265.5 L / dry metric ton feedstock
Feedstock Cost	$88.18 /dry metric ton
Internal Rate of Return (After-Tax)	10%
Equity Percent of Total Investment	40%

Capital Costs		
RCF Area (Reactor Only Costs)		$173,948,328
RCF Area (Non-Reactor Costs)		$15,880,435
Saccharification & Fermentation		$21,800,000
On-site Enzyme Production		$25,000,000
Distillation and Solids Recovery		$19,100,000
Wastewater Treatment		$42,600,000
Storage		$3,900,000
Boiler/Turbogenerator		$116,300,000
Utilities		$9,900,000
Total Installed Equipment Cost		$428,428,763
Added Direct + Indirect Costs		$368,271,237
(% of TCI)	46%	
Total Capital Investment (TCI)		$796,700,000
Lignin Yield (kg/dry metric ton)		200.8
(wt.% biomass lignin)	70.3%	
Monomer Yield (kg/dry metric ton)		35.6
(wt.% biomass lignin)	12.5%	
Maximum Ethanol Yields (100% of Theoretical)		
Ethanol Production (MML/yr)		310.2
Theoretical Yield (L/dry metric ton)		472.2
Current Yield (Actual/Theoretical)		56.2%
RCF Area Heat Demand (MJ/s)		307.3
(% biomass energy LHV)		75.8%
Natural Gas Imports (kg/kg crude RCF oil)		1.03
Excess Electricity (kWh/kg crude RCF oil)		1.3
Plant Water Usage (L/kg crude RCF oil)		6.2

RCF Oil Composition (wt.%)	
Total Lignin	56.7%
Monomers	10.0%
Dimers	35.0%
Oligomers	11.7%
Carbohydrate Derivatives	38.0%
Extractives	4.4%
Residual Solvent/Water	1.0%

Manufacturing Costs (cents/kg crude RCF oil)	
Feedstock	24.9
Methanol	0.0
Hydrogen	0.0
Glucose (enzyme production)	9.0
Catalysts	1.6
Other Raw Materials	2.4
Natural Gas	27.0
Waste Disposal	0.2
Net Electricity	-7.8
Ethanol	-32.6
Fixed Costs	0.0
Capital Depreciation	10.8
Average Income Tax	2.2
Average Return on Investment	38.6

Manufacturing Costs ($/yr)	
Feedstock	$57,900,000
Methanol	$0
Hydrogen	$0
Glucose (enzyme production)	$20,900,000
Catalysts	$3,700,000
Other Raw Materials	$5,600,000
Natural Gas	$62,800,000
Waste Disposal	$400,000
Net Electricity	-$18,200,000
Ethanol	-$75,800,000
Fixed Costs	$18,400,000
Capital Depreciation	$25,200,000
Average Income Tax	$5,000,000
Average Return on Investment	$89,700,000

RCF Specific Operating Conditions	
RCF Reactor Temperature (°C)	210
RCF Reactor Pressure (bar)	30
RCF Residence Time (h)	2
Reactor Solvent Loading (L/dry kg biomass)	9
Biomass Delignification	75%
RCF Reactor Total Solids	11.7%
Net RCF Oil Recovery	93.0%

Table S16. Ethylene Glycol Case Summary.

Ethylene Glycol Case Summary

(All Values in 2016$)

MSP-Crude RCF Oil	**$0.98 /kg**
MSP-Lignin Fraction	**$1.51 /kg**
MSP-Monomer Fraction	**$3.07 /kg**
Ethanol Selling Price	**$1.65 /gal**
Crude RCF Oil Production	192.1 MMkg/yr
Crude RCF Oil Yield	292.4 kg/dry metric ton
Ethanol Production	206.3 MML/yr (Ethanol at 68 °F)
Ethanol Yield	314.0 L / dry metric ton feedstock
Feedstock Cost	$88.18 /dry metric ton
Internal Rate of Return (After-Tax)	10%
Equity Percent of Total Investment	40%

Capital Costs	
RCF Area (Reactor Only Costs)	$122,737,325
RCF Area (Non-Reactor Costs)	$40,070,897
Saccharification & Fermentation	$23,700,000
On-site Enzyme Production	$23,200,000
Distillation and Solids Recovery	$21,000,000
Wastewater Treatment	$43,700,000
Storage	$4,300,000
Boiler/Turbogenerator	$119,100,000
Utilities	$10,400,000
Total Installed Equipment Cost	$408,208,222
Added Direct + Indirect Costs	$347,291,778
(% of TCI)	46%
Total Capital Investment (TCI)	$755,500,000
Lignin Yield (kg/dry metric ton)	190.7
(wt.% biomass lignin)	66.8%
Monomer Yield (kg/dry metric ton)	93.4
(wt.% biomass lignin)	32.7%
Maximum Ethanol Yields (100% of Theoretical)	
Ethanol Production (MML/yr)	310.2
Theoretical Yield (L/dry metric ton)	472.2
Current Yield (Actual/Theoretical)	66.5%
RCF Area Heat Demand (MJ/s)	316.8
(% biomass energy LHV)	78.2%
Natural Gas Imports (kg/kg crude RCF oil)	1.26
Excess Electricity (kWh/kg crude RCF oil)	1.7
Plant Water Usage (L/kg crude RCF oil)	9.1

RCF Oil Composition (wt.%)	
Total Lignin	65.2%
Monomers	32.0%
Dimers	16.6%
Oligomers	16.6%
Carbohydrate Derivatives	29.0%
Extractives	5.4%
Residual Solvent/Water	0.0%

Manufacturing Costs (cents/kg crude RCF oil)	
Feedstock	30.2
Ethylene Glycol	16.3
Hydrogen	2.2
Glucose (enzyme production)	10.1
Catalysts	0.5
Other Raw Materials	2.9
Natural Gas	32.8
Waste Disposal	0.2
Net Electricity	-9.7
Ethanol	-46.7
Fixed Costs	0.0
Capital Depreciation	12.4
Average Income Tax	2.4
Average Return on Investment	44.6

Manufacturing Costs ($/yr)	
Feedstock	$57,900,000
Ethylene Glycol	$31,300,000
Hydrogen	$4,300,000
Glucose (enzyme production)	$19,400,000
Catalysts	$900,000
Other Raw Materials	$5,600,000
Natural Gas	$63,000,000
Waste Disposal	$400,000
Net Electricity	-$18,600,000
Ethanol	-$89,700,000
Fixed Costs	$17,400,000
Capital Depreciation	$23,900,000
Average Income Tax	$4,700,000
Average Return on Investment	$85,500,000

RCF Specific Operating Conditions	
RCF Reactor Temperature (°C)	200
RCF Reactor Pressure (bar)	6
RCF Residence Time (h)	3
Reactor Solvent Loading (L/dry kg biomass)	9
Biomass Delignification	70%
RCF Reactor Total Solids	9.5%
Net RCF Oil Recovery	95.6%

Table S17. Tabulated data from main text Figure 5a.

Component	Crude RCF Oil Yield (kg/dry metric ton feedstock)				Crude RCF Oil Production (MMkg/yr)			
	Methanol Case	Ethanol Case	Hydrogen-Free Case	Ethylene Glycol Case	Methanol Case	Ethanol Case	Hydrogen-Free Case	Ethylene Glycol Case
All Lignin Components	183.7	162.4	200.8	191.4	120.7	106.8	131.9	125.6
Monomers	88.4	81.0	35.6	93.4	58.1	53.3	23.4	61.4
Dimers	47.6	40.7	123.9	48.9	31.3	26.8	81.4	32.1
Oligomers	47.6	40.7	41.3	49.0	31.3	26.8	27.1	32.1
Carbohydrate Derivatives	82.5	79.1	134.6	85.4	54.2	52.0	88.3	56.0
Extractives	15.3	15.2	15.5	15.8	10.0	10.0	10.1	10.4
Water	1.35	1.18	3.56	0	0.89	0.78	2.34	0.00
Methanol	0.22	0	0	0	0.15	0	0	0
Ethanol	0	0.03	0.01	0	0	0.02	0.004	0
Ethylene Glycol	0	0	0	1.11	0	0	0	0.73
Total	283.0	257.9	354.3	294.2	185.9	169.4	232.8	193.1

Table S18. Tabulated data from main text Figure 5b. Costs are in MM USD. Additional capital cost data may be found in case summary tables (**Table S4, Tables S14-S16).**

Process Area	Methanol Case	Ethanol Case	Hydrogen-Free Case	Ethylene Glycol Case
RCF	323.5	287.5	189.8	162.8
Saccharification & Fermentation	23.7	24.1	21.8	23.7
On-site Enzyme Production	23.2	25.0	25.0	23.2
Distillation and Solids Recovery	21	20.8	19.1	21.0
Wastewater Treatment	43.5	45.4	42.6	43.7
Storage	4.4	3.9	3.9	4.3
Boiler/Turbogenerator	114.4	106.7	116.3	119.1
Utilities	10	9.5	9.9	10.4
Total Installed Equipment Cost	563.7	522.9	428.4	408.2

Table S19. Tabulated data from main text Figure 5c. Costs are in MM USD/yr. Additional operating cost data may be found in case summary tables (**Table S4, Tables S14-S16).**

Operating Cost	Methanol Case	Ethanol Case	Hydrogen-Free Case	Ethylene Glycol Case
Other	25.2	26.4	26.9	25.4
Feedstock	57.9	57.9	57.9	57.9
Natural Gas	55.0	48.0	62.8	63.0
RCF Solvent Makeup	12.1	0	0	31.3
Hydrogen for RCF	5.5	3.3	0	4.3
Catalyst	0.9	5.6	3.7	0.9
Total Variable Operating Cost	156.7	141.2	151.4	182.8

Table S20. Tabulated data from main text Figure 5d. Units for GWP-Lignin Fraction are kg CO_2-eq/kg.

Process Input	Methanol Case	Ethanol Case	Hydrogen-Free Case	Ethylene Glycol Case
Process Emissions: Other Sources	2.00	2.45	1.81	1.87
Process Emissions: Natural Gas	2.04	2.07	2.45	2.30
Catalyst	3.48E-04	4.05E-04	2.44E-04	3.42E-04
Hydrogen for RCF	0.02	0.01	0.00	0.02
RCF Solvent Makeup	0.11	0.00	0.00	0.23
Natural Gas	0.75	0.76	0.90	0.84
Feedstock	-4.13	-4.81	-4.34	-4.06
Other	-0.72	-0.67	-0.84	-0.81
Net GWP-Lignin Fraction	0.08	-0.18	-0.02	0.39

Table S21. Tabulated data from main text Figure 5e. Units for CED-Lignin Fraction are MJ/kg.

Process Input	Methanol Case	Ethanol Case	Hydrogen-Free Case	Ethylene Glycol Case
Catalyst	0.006	0.007	0.004	0.006
Hydrogen for RCF	0.85	0.59	0	0.65
RCF Solvent Makeup	5.14	0	0	7.03
Natural Gas	35.46	36.07	42.53	39.96
Feedstock	42.89	49.99	45.03	42.17
Other	-10.32	-9.18	-12.22	-11.89
Net CED-Lignin Fraction	74.03	77.47	75.36	77.93

Table S22. GWP-Lignin Fraction and CED-Lignin Fraction for each configuration, economic allocation.

	Methanol Case	Ethanol Case	Hydrogen-Free Case	Ethylene Glycol Case
GWP (kg CO_2-eq/kg)	0.13	-0.28	-0.02	0.64
CED (MJ/kg)	122.05	125.47	115.73	126.84

Table S23. Tabulated data from main text Figure 6.

	**Maximum Membrane Capital Cost Allowance ($/[L/hr])**		
**RCF Process Configuration**	Ethanol Selling Price at $1.65/gal	Ethanol Selling Price at $1.97/gal	Ethanol Selling Price at $2.30/gal
Ethanol Case (MSP-Crude RCF Oil = $1.18/kg)	346	416	486
Methanol Case (MSP-Crude RCF Oil = $1.13/kg)	310	380	450
Ethylene Glycol Case (MSP-Crude RCF Oil = $0.98/kg)	198	268	338
Hydrogen-Free Case (MSP-Crude RCF Oil = $0.76/kg)	32	102	172

Table S24. Methanol case non-GHG emissions.

Source Description	CO (tpy)	NO_x (tpy)	PM (filterable) (tpy)	PM_{10} (tpy)	$PM_{2.5}$ (tpy)	SO_2 (tpy)	VOC (tpy)
Feed Handling			7.66	7.66	1.30		
RCF Process							
Enzymatic Hydrolysis and Fermentation							41.69
Enzyme Production						2.11	
Ethanol Recovery							
WWT							5.84
Tanks							46.20
Loading Operations							73.24
Boiler	746.53	559.90	0.17	1.18	1.18	23.69	48.88
Emergency Generator	0.43	0.50	2.47E-02	2.47E-02	2.47E-02	8.15E-04	0.50
Emergency Fire Pump	0.25	0.21	1.52E-02	1.52E-02	1.52E-02	3.40E-04	0.21
Cooling Tower			2.36	1.65	0.99		214.77
Equipment Leaks							42.81
Truck Traffic			75.12	20.56	2.55		
Total Emissions	**747**	**561**	**85**	**31**	**6**	**26**	**474**

Table S25. Ethanol case non-GHG emissions.

Source Description	CO (tpy)	NO_x (tpy)	PM (filterable) (tpy)	PM_{10} (tpy)	$PM_{2.5}$ (tpy)	SO_2 (tpy)	VOC (tpy)
Feed Handling			7.66	7.66	1.30		
RCF Process							
Enzymatic Hydrolysis and Fermentation							41.66
Enzyme Production						2.23	
Ethanol Recovery							
WWT							0.00
Tanks							26.87
Loading Operations							43.26
Boiler	663.78	497.83	0.15	1.05	1.05	24.63	43.46
Emergency Generator	0.43	0.50	2.47E-02	2.47E-02	2.47E-02	8.15E-04	0.50
Emergency Fire Pump	0.25	0.21	1.52E-02	1.52E-02	1.52E-02	3.40E-04	0.21
Cooling Tower			2.05	1.44	0.86		186.47
Equipment Leaks							36.73
Truck Traffic			72.14	19.75	2.45		
Total Emissions	**664**	**499**	**82**	**30**	**6**	**27**	**379**

Table S26. Hydrogen-free case non-GHG emissions.

Source Description	CO (tpy)	NO_x (tpy)	PM (filterable) (tpy)	PM_{10} (tpy)	$PM_{2.5}$ (tpy)	SO_2 (tpy)	VOC (tpy)
Feed Handling			7.66	7.66	1.30		
RCF Process							
Enzymatic Hydrolysis and Fermentation							41.66
Enzyme Production						2.28	
Ethanol Recovery							
WWT							0.00
Tanks							27.09
Loading Operations							43.64
Boiler	756.76	567.57	0.17	1.20	1.20	24.58	49.55
Emergency Generator	0.43	0.50	2.47E-02	2.47E-02	2.47E-02	8.15E-04	0.50
Emergency Fire Pump	0.25	0.21	1.52E-02	1.52E-02	1.52E-02	3.40E-04	0.21
Cooling Tower			2.25	1.58	0.95		204.83
Equipment Leaks							42.21
Truck Traffic			75.31	20.61	2.56		
Total Emissions	**757**	**568**	**85**	**31**	**6**	**27**	**410**

Table S27. Ethylene glycol case non-GHG emissions.

Source Description	CO (tpy)	NO_x (tpy)	PM (filterable) (tpy)	PM_{10} (tpy)	$PM_{2.5}$ (tpy)	SO_2 (tpy)	VOC (tpy)
Feed Handling			7.66	7.66	1.30		
RCF Process							
Enzymatic Hydrolysis and Fermentation							41.66
Enzyme Production						2.11	
Ethanol Recovery							
WWT							5.78
Tanks							31.97
Loading Operations							51.61
Boiler	800.55	600.41	0.18	1.27	1.27	23.37	52.42
Emergency Generator	0.43	0.50	2.47E-02	2.47E-02	2.47E-02	8.15E-04	0.50
Emergency Fire Pump	0.25	0.21	1.52E-02	1.52E-02	1.52E-02	3.40E-04	0.21
Cooling Tower			2.58	1.81	1.08		234.74
Equipment Leaks							43.23
Truck Traffic			75.94	20.79	2.58		
Total Emissions	**801**	**601**	**86**	**32**	**6**	**25**	**462**

Table S28. Ethylene glycol membrane case non-GHG emissions.

Source Description	CO (tpy)	NO_x (tpy)	PM (filterable) (tpy)	PM_{10} (tpy)	$PM_{2.5}$ (tpy)	SO_2 (tpy)	VOC (tpy)
Feed Handling			7.66	7.66	1.30		
RCF Process							
Enzymatic Hydrolysis and Fermentation							41.66
Enzyme Production						2.11	
Ethanol Recovery							
WWT							4.84
Tanks							31.97
Loading Operations							51.61
Boiler	317.13	237.85	0.07	0.50	0.50	23.36	20.76
Emergency Generator	0.43	0.50	2.47E-02	2.47E-02	2.47E-02	8.15E-04	0.50
Emergency Fire Pump	0.25	0.21	1.52E-02	1.52E-02	1.52E-02	3.40E-04	0.21
Cooling Tower			1.00	0.70	0.42		90.76
Equipment Leaks							43.21
Truck Traffic			70.84	19.39	2.40		
Total Emissions	**318**	**239**	**80**	**28**	**5**	**25**	**286**

Table S29. Estimated hazardous air pollutants from the methanol case.

HAP Pollutant	Enzymatic Hydrolysis and Fermentation (tpy)	WWT (tpy)	Tanks (tpy)	Loading Operations (tpy)	Boiler (tpy)	Emergency Generator (tpy)	Emergency Fire Pump (tpy)	Cooling Tower (tpy)	Equipment Leaks (tpy)	Facility-Wide HAPs Total (tpy)
1,3-Butadiene						2.05E-05	8.55E-06			2.91E-05
Acenaphthene					1.60E-05	7.46E-07	3.11E-07			1.71E-05
Acenaphthylene					1.60E-05	2.66E-06	1.11E-06			1.98E-05
Acetaldehyde						4.03E-04	1.68E-04			5.70E-04
2-Methylnaphthalene					2.18E-04					2.18E-04
Acrolein						4.86E-05	2.02E-05			6.88E-05
Anthracene					2.13E-05	9.82E-07	4.09E-07			2.27E-05
3-Methylcholanthrene					1.60E-05					1.60E-05
7,12-Dimethylben(a)anthracene					1.42E-04					1.42E-04
Benzene					1.87E-02	4.90E-04	2.04E-04			1.94E-02
Benzo(a)anthracene					1.60E-05	8.82E-07	3.68E-07			1.72E-05
Benzo(a)pyrene					1.07E-05	9.87E-08	4.11E-08			1.08E-05
Benzo(b)fluoranthene					1.60E-05	5.20E-08	2.17E-08			1.61E-05
Benzo(g,h,i)perylene					1.07E-05	2.57E-07	1.07E-07			1.10E-05
Benzo(k)fluoranthene					1.60E-05	8.14E-08	3.39E-08			1.61E-05
Biphenyl										0.00E+00
Cadmium Metal (elemental un-reacted) –(Add w/CDC)										0.00E+00
Butane					1.87E+01					1.87E+01
Chromium–Other compds (add w/chrom acid to get CRC)										0.00E+00
Chrysene					1.60E-05	1.85E-07	7.72E-08			1.63E-05
Cumene										0.00E+00
Dibenzo(a,h)anthracene					1.07E-05	3.06E-07	1.28E-07			1.11E-05
Dichlorobenzene					1.07E-02					1.07E-02
Ethane					2.76E+01					2.76E+01
Ethyl benzene										0.00E+00
Fluoranthene					3.20E-05	4.00E-06	1.66E-06			3.77E-05
Fluorene					2.49E-05	1.53E-05	6.39E-06			4.66E-05
Formaldehyde					6.67E-01	6.20E-04	2.58E-04			6.67E-01
Hexachlorodibenzo-p-dioxin 1,2,3,6,7,8										0.00E+00
Hexane					1.60E+01					1.60E+01
Indeno(1,2,3-cd)pyrene					1.60E-02	1.97E-07	8.20E-08			1.60E-02
Lead and Lead compounds										0.00E+00
Manganese & compounds										0.00E+00
Methanol	0.02	5.84	13.59	2.16E+01						4.10E+01
Naphthalene					5.42E-03	4.45E-05	1.86E-05			5.48E-03
Nickel metal (Component of Nickel & Compounds)										0.00E+00
Pentane					2.31E+01					2.31E+01
Phenanthrene					1.51E-04	1.54E-05	6.43E-06			1.73E-04
Propylene						1.50E-05	6.23E-06			2.12E-05
Pyrene					4.44E-05	2.51E-06	1.05E-06			4.80E-05
Propane					1.42E+01					1.42E+01
Toluene					3.02E-02	2.15E-04	8.95E-05			3.05E-02
Trimethyl pentane 2,2,4-										0.00E+00
Xylene (Total)						1.50E-04	6.23E-05			2.12E-04
Unknown			3.50E-07					1.07E+02	2.14E+01	1.29E+02
**Total Pollutants**	**2.32E-02**	**5.84E+00**	**1.36E+01**	**2.16E+01**	**1.00E+02**	**2.05E-03**	**8.54E-04**	**1.07E+02**	**2.14E+01**	**270.11**

Table S30. Estimated hazardous air pollutants from the ethanol case.

HAP Pollutant	Enzymatic Hydrolysis and Fermentation (tpy)	WWT (tpy)	Tanks (tpy)	Loading Operations (tpy)	Boiler (tpy)	Emergency Generator (tpy)	Emergency Fire Pump (tpy)	Cooling Tower (tpy)	Equipment Leaks (tpy)	Facility-Wide HAPs Total (tpy)
1,3-Butadiene						2.05E-05	8.55E-06			2.91E-05
Acenaphthene					1.42E-05	7.46E-07	3.11E-07			1.53E-05
Acenaphthylene					1.42E-05	2.66E-06	1.11E-06			1.80E-05
Acetaldehyde						4.03E-04	1.68E-04			5.70E-04
2-Methylnaphthalene					1.94E-04					1.94E-04
Acrolein						4.86E-05	2.02E-05			6.88E-05
Anthracene					1.90E-05	9.82E-07	4.09E-07			2.04E-05
3-Methylcholanthrene					1.42E-05					1.42E-05
7,12-Dimethylben(a)anthracene					1.26E-04					1.26E-04
Benzene					1.66E-02	4.90E-04	2.04E-04			1.73E-02
Benzo(a)anthracene					1.42E-05	8.82E-07	3.68E-07			1.55E-05
Benzo(a)pyrene					9.48E-06	9.87E-08	4.11E-08			9.62E-06
Benzo(b)fluoranthene					1.42E-05	5.20E-08	2.17E-08			1.43E-05
Benzo(g,h,i)perylene					9.48E-06	2.57E-07	1.07E-07			9.85E-06
Benzo(k)fluoranthene					1.42E-05	8.14E-08	3.39E-08			1.43E-05
Biphenyl										0.00E+00
Cadmium Metal (elemental un-reacted) –(Add w/CDC)										0.00E+00
Butane					1.66E+01					1.66E+01
Chromium–Other compds (add w/chrom acid to get CRC)										0.00E+00
Chrysene					1.42E-05	1.85E-07	7.72E-08			1.45E-05
Cumene										0.00E+00
Dibenzo(a,h)anthracene					9.48E-06	3.06E-07	1.28E-07			9.92E-06
Dichlorobenzene					9.48E-03					9.48E-03
Ethane					2.45E+01					2.45E+01
Ethyl benzene										0.00E+00
Fluoranthene					2.84E-05	4.00E-06	1.66E-06			3.41E-05
Fluorene					2.21E-05	1.53E-05	6.39E-06			4.38E-05
Formaldehyde					5.93E-01	6.20E-04	2.58E-04			5.94E-01
Hexachlorodibenzo-p-dioxin 1,2,3,6,7,8										0.00E+00
Hexane					1.42E+01					1.42E+01
Indeno(1,2,3-cd)pyrene					1.42E-02	1.97E-07	8.20E-08			1.42E-02
Lead and Lead compounds										0.00E+00
Manganese & compounds										0.00E+00
Methanol										0.00E+00
Naphthalene					4.82E-03	4.45E-05	1.86E-05			4.88E-03
Nickel metal (Component of Nickel & Compounds)										0.00E+00
Pentane					2.05E+01					2.05E+01
Phenanthrene					1.34E-04	1.54E-05	6.43E-06			1.56E-04
Propylene						1.50E-05	6.23E-06			2.12E-05
Pyrene					3.95E-05	2.51E-06	1.05E-06			4.31E-05
Propane					1.26E+01					1.26E+01
Toluene					2.69E-02	2.15E-04	8.95E-05			2.72E-02
Trimethyl pentane 2,2,4-										0.00E+00
Xylene (Total)						1.50E-04	6.23E-05			2.12E-04
Unknown			3.50E-07					9.32E+01	1.84E+01	1.12E+02
**Total Pollutants**	**0.00E+00**	**0.00E+00**	**3.50E-07**	**0.00E+00**	**8.92E+01**	**2.05E-03**	**8.54E-04**	**9.32E+01**	**1.84E+01**	**200.77**

Table S31. Estimated hazardous air pollutants from the hydrogen-free case.

HAP Pollutant	Enzymatic Hydrolysis and Fermentation (tpy)	WWT (tpy)	Tanks (tpy)	Loading Operations (tpy)	Boiler (tpy)	Emergency Generator (tpy)	Emergency Fire Pump (tpy)	Cooling Tower (tpy)	Equipment Leaks (tpy)	Facility-Wide HAPs Total (tpy)
1,3-Butadiene						2.05E-05	8.55E-06			2.91E-05
Acenaphthene					1.62E-05	7.46E-07	3.11E-07			1.73E-05
Acenaphthylene					1.62E-05	2.66E-06	1.11E-06			2.00E-05
Acetaldehyde						4.03E-04	1.68E-04			5.70E-04
2-Methylnaphthalene					2.21E-04					2.21E-04
Acrolein						4.86E-05	2.02E-05			6.88E-05
Anthracene					2.16E-05	9.82E-07	4.09E-07			2.30E-05
3-Methylcholanthrene					1.62E-05					1.62E-05
7,12-Dimethylben(a)anthracene					1.44E-04					1.44E-04
Benzene					1.89E-02	4.90E-04	2.04E-04			1.96E-02
Benzo(a)anthracene					1.62E-05	8.82E-07	3.68E-07			1.75E-05
Benzo(a)pyrene					1.08E-05	9.87E-08	4.11E-08			1.10E-05
Benzo(b)fluoranthene					1.62E-05	5.20E-08	2.17E-08			1.63E-05
Benzo(g,h,i)perylene					1.08E-05	2.57E-07	1.07E-07			1.12E-05
Benzo(k)fluoranthene					1.62E-05	8.14E-08	3.39E-08			1.63E-05
Biphenyl										0.00E+00
Cadmium Metal (elemental un-reacted) –(Add w/CDC)										0.00E+00
Butane					1.89E+01					1.89E+01
Chromium–Other compds (add w/chrom acid to get CRC)										0.00E+00
Chrysene					1.62E-05	1.85E-07	7.72E-08			1.65E-05
Cumene										0.00E+00
Dibenzo(a,h)anthracene					1.08E-05	3.06E-07	1.28E-07			1.12E-05
Dichlorobenzene					1.08E-02					1.08E-02
Ethane					2.79E+01					2.79E+01
Ethyl benzene										0.00E+00
Fluoranthene					3.24E-05	4.00E-06	1.66E-06			3.81E-05
Fluorene					2.52E-05	1.53E-05	6.39E-06			4.69E-05
Formaldehyde					6.76E-01	6.20E-04	2.58E-04			6.77E-01
Hexachlorodibenzo-p-dioxin 1,2,3,6,7,8										0.00E+00
Hexane					1.62E+01					1.62E+01
Indeno(1,2,3-cd)pyrene					1.62E-02	1.97E-07	8.20E-08			1.62E-02
Lead and Lead compounds										0.00E+00
Manganese & compounds										0.00E+00
Methanol										0.00E+00
Naphthalene					5.50E-03	4.45E-05	1.86E-05			5.56E-03
Nickel metal (Component of Nickel & Compounds)										0.00E+00
Pentane					2.34E+01					2.34E+01
Phenanthrene					1.53E-04	1.54E-05	6.43E-06			1.75E-04
Propylene						1.50E-05	6.23E-06			2.12E-05
Pyrene					4.50E-05	2.51E-06	1.05E-06			4.86E-05
Propane					1.44E+01					1.44E+01
Toluene					3.06E-02	2.15E-04	8.95E-05			3.09E-02
Trimethyl pentane 2,2,4-										0.00E+00
Xylene (Total)						1.50E-04	6.23E-05			2.12E-04
Unknown			4.50E-07					1.02E+02	2.11E+01	1.24E+02
**Total Pollutants**	**0.00E+00**	**0.00E+00**	**4.50E-07**	**0.00E+00**	**1.02E+02**	**2.05E-03**	**8.54E-04**	**1.02E+02**	**2.11E+01**	**225.18**

Table S32. Estimated hazardous air pollutants from the ethylene glycol case.

HAP Pollutant	Enzymatic Hydrolysis and Fermentation (tpy)	WWT (tpy)	Tanks (tpy)	Loading Operations (tpy)	Boiler (tpy)	Emergency Generator (tpy)	Emergency Fire Pump (tpy)	Cooling Tower (tpy)	Equipment Leaks (tpy)	Facility-Wide HAPs Total (tpy)
1,3-Butadiene						2.05E-05	8.55E-06			2.91E-05
Acenaphthene					1.72E-05	7.46E-07	3.11E-07			1.82E-05
Acenaphthylene					1.72E-05	2.66E-06	1.11E-06			2.09E-05
Acetaldehyde						4.03E-04	1.68E-04			5.70E-04
2-Methylnaphthalene					2.33E-04					2.33E-04
Acrolein						4.86E-05	2.02E-05			6.88E-05
Anthracene					2.29E-05	9.82E-07	4.09E-07			2.43E-05
3-Methylcholanthrene					1.72E-05					1.72E-05
7,12-Dimethylben(a)anthracene					1.52E-04					1.52E-04
Benzene					2.00E-02	4.90E-04	2.04E-04			2.07E-02
Benzo(a)anthracene					1.72E-05	8.82E-07	3.68E-07			1.84E-05
Benzo(a)pyrene					1.14E-05	9.87E-08	4.11E-08			1.16E-05
Benzo(b)fluoranthene					1.72E-05	5.20E-08	2.17E-08			1.72E-05
Benzo(g,h,i)perylene					1.14E-05	2.57E-07	1.07E-07			1.18E-05
Benzo(k)fluoranthene					1.72E-05	8.14E-08	3.39E-08			1.73E-05
Biphenyl										0.00E+00
Cadmium Metal (elemental un-reacted) –(Add w/CDC)										0.00E+00
Butane					2.00E+01					2.00E+01
Chromium–Other compds (add w/chrom acid to get CRC)										0.00E+00
Chrysene					1.72E-05	1.85E-07	7.72E-08			1.74E-05
Cumene										0.00E+00
Dibenzo(a,h)anthracene					1.14E-05	3.06E-07	1.28E-07			1.19E-05
Dichlorobenzene					1.14E-02					1.14E-02
Ethane					2.95E+01					2.95E+01
Ethyl benzene										0.00E+00
Fluoranthene					3.43E-05	4.00E-06	1.66E-06			4.00E-05
Fluorene					2.67E-05	1.53E-05	6.39E-06			4.84E-05
Formaldehyde					7.15E-01	6.20E-04	2.58E-04			7.16E-01
Hexachlorodibenzo-p-dioxin 1,2,3,6,7,8										0.00E+00
Hexane					1.72E+01					1.72E+01
Indeno(1,2,3-cd)pyrene					1.72E-02	1.97E-07	8.20E-08			1.72E-02
Lead and Lead compounds										0.00E+00
Manganese & compounds										0.00E+00
Methanol										0.00E+00
Naphthalene					5.81E-03	4.45E-05	1.86E-05			5.88E-03
Nickel metal (Component of Nickel & Compounds)										0.00E+00
Pentane					2.48E+01					2.48E+01
Phenanthrene					1.62E-04	1.54E-05	6.43E-06			1.84E-04
Propylene						1.50E-05	6.23E-06			2.12E-05
Pyrene					4.77E-05	2.51E-06	1.05E-06			5.12E-05
Propane					1.52E+01					1.52E+01
Toluene					3.24E-02	2.15E-04	8.95E-05			3.27E-02
Trimethyl pentane 2,2,4-										0.00E+00
Xylene (Total)						1.50E-04	6.23E-05			2.12E-04
Unknown			4.00E-07					1.17E+02	2.16E+01	1.39E+02
**Total Pollutants**	**0.00E+00**	**0.00E+00**	**4.00E-07**	**0.00E+00**	**1.08E+02**	**2.05E-03**	**8.54E-04**	**1.17E+02**	**2.16E+01**	**246.53**

Table S33. Estimated hazardous air pollutants from the ethylene glycol membrane case.

HAP Pollutant	Enzymatic Hydrolysis and Fermentation (tpy)	WWT (tpy)	Tanks (tpy)	Loading Operations (tpy)	Boiler (tpy)	Emergency Generator (tpy)	Emergency Fire Pump (tpy)	Cooling Tower (tpy)	Equipment Leaks (tpy)	Facility-Wide HAPs Total (tpy)
1,3-Butadiene						2.05E-05	8.55E-06			2.91E-05
Acenaphthene					6.80E-06	7.46E-07	3.11E-07			7.85E-06
Acenaphthylene					6.80E-06	2.66E-06	1.11E-06			1.06E-05
Acetaldehyde						4.03E-04	1.68E-04			5.70E-04
2-Methylnaphthalene					9.25E-05					9.25E-05
Acrolein						4.86E-05	2.02E-05			6.88E-05
Anthracene					9.06E-06	9.82E-07	4.09E-07			1.05E-05
3-Methylcholanthrene					6.80E-06					6.80E-06
7,12-Dimethylben(a)anthracene					6.04E-05					6.04E-05
Benzene					7.93E-03	4.90E-04	2.04E-04			8.62E-03
Benzo(a)anthracene					6.80E-06	8.82E-07	3.68E-07			8.05E-06
Benzo(a)pyrene					4.53E-06	9.87E-08	4.11E-08			4.67E-06
Benzo(b)fluoranthene					6.80E-06	5.20E-08	2.17E-08			6.87E-06
Benzo(g,h,i)perylene					4.53E-06	2.57E-07	1.07E-07			4.89E-06
Benzo(k)fluoranthene					6.80E-06	8.14E-08	3.39E-08			6.91E-06
Biphenyl										0.00E+00
Cadmium Metal (elemental un-reacted) –(Add w/CDC)										0.00E+00
Butane					7.93E+00					7.93E+00
Chromium–Other compds (add w/chrom acid to get CRC)										0.00E+00
Chrysene					6.80E-06	1.85E-07	7.72E-08			7.06E-06
Cumene										0.00E+00
Dibenzo(a,h)anthracene					4.53E-06	3.06E-07	1.28E-07			4.96E-06
Dichlorobenzene					4.53E-03					4.53E-03
Ethane					1.17E+01					1.17E+01
Ethyl benzene										0.00E+00
Fluoranthene					1.36E-05	4.00E-06	1.66E-06			1.93E-05
Fluorene					1.06E-05	1.53E-05	6.39E-06			3.23E-05
Formaldehyde					2.83E-01	6.20E-04	2.58E-04			2.84E-01
Hexachlorodibenzo-p-dioxin 1,2,3,6,7,8										0.00E+00
Hexane					6.80E+00					6.80E+00
Indeno(1,2,3-cd)pyrene					6.80E-03	1.97E-07	8.20E-08			6.80E-03
Lead and Lead compounds										0.00E+00
Manganese & compounds										0.00E+00
Methanol										0.00E+00
Naphthalene					2.30E-03	4.45E-05	1.86E-05			2.37E-03
Nickel metal (Component of Nickel & Compounds)										0.00E+00
Pentane					9.82E+00					9.82E+00
Phenanthrene					6.42E-05	1.54E-05	6.43E-06			8.60E-05
Propylene						1.50E-05	6.23E-06			2.12E-05
Pyrene					1.89E-05	2.51E-06	1.05E-06			2.24E-05
Propane					6.04E+00					6.04E+00
Toluene					1.28E-02	2.15E-04	8.95E-05			1.31E-02
Trimethyl pentane 2,2,4-										0.00E+00
Xylene (Total)						1.50E-04	6.23E-05			2.12E-04
Unknown			4.00E-07					4.54E+01	2.16E+01	6.70E+01
**Total Pollutants**	**0.00E+00**	**0.00E+00**	**4.00E-07**	**0.00E+00**	**4.26E+01**	**2.05E-03**	**8.54E-04**	**4.54E+01**	**2.16E+01**	**109.59**

Table S34. Tabulated data from main text Figure 8. Data taken from Reference 9.

Product	US Price ($/lb)	US Price ($/kg)	Global Consumption (MM metric tons)
Acetic Acid	0.33	0.73	12.92
Acetone	0.77	1.69	6.75
Acrylic Acid	1.62	3.58	12.03
Acrylonitrile	1.21	2.67	6.30
Adipic Acid	1.03	2.28	2.46
Benzene	0.57	1.26	18.93
Butadiene (1,3-)	1.32	2.90	10.56
Butanediol (1,4-)	1.61	3.55	1.56
Epichlorohydrin	0.91	2.00	1.10
Ethanol	0.30	0.67	77.53
Ethyl Acetate	1.22	2.70	1.79
Ethyl Lactate	0.86	1.90	1.20
Ethylene	0.45	1.00	122.90
Fatty Acids	0.52	1.15	6.01
Furfural	0.73	1.60	0.27
Refined Glycerin	0.50	1.10	2.00
Isoprene	1.04	2.30	0.87
Lactic Acid	0.77	1.70	0.48
Lipids	0.53	1.17	166.93
Methanol	0.21	0.47	59.53
Oxo Chemicals	1.26	2.77	12.19
PHA	0.68	1.50	0.41
Gasoline	0.57	1.25	929.81
Diesel	0.52	1.15	1291.18
Phenol	0.66	1.46	8.90
Propylene Glycols	0.63	1.38	1.64
Sorbitol	0.39	0.85	1.88
Succinic Acid	1.13	2.50	0.05
THF	1.20	2.64	0.62
Xylene (para)	0.74	1.64	32.07
Xylitol	2.05	4.51	0.90

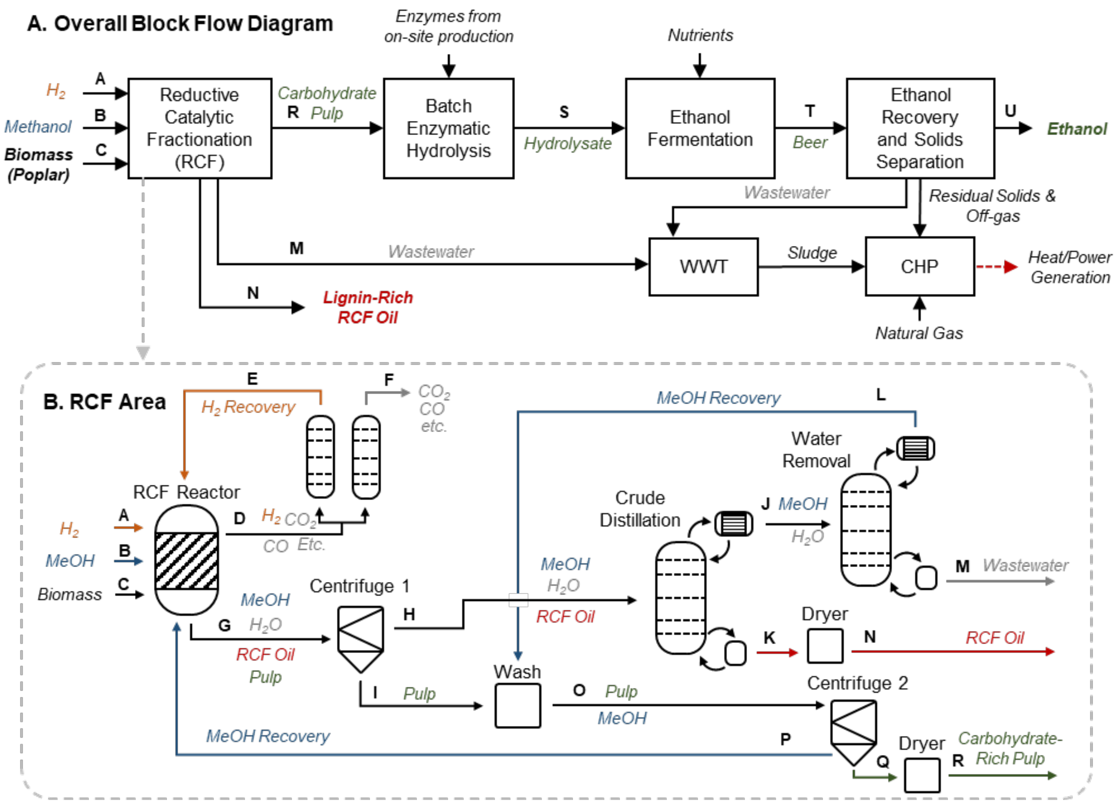

Figure S1. **Methanol case process flow diagram with stream labels. Reference Table S2 for stream compositions.**

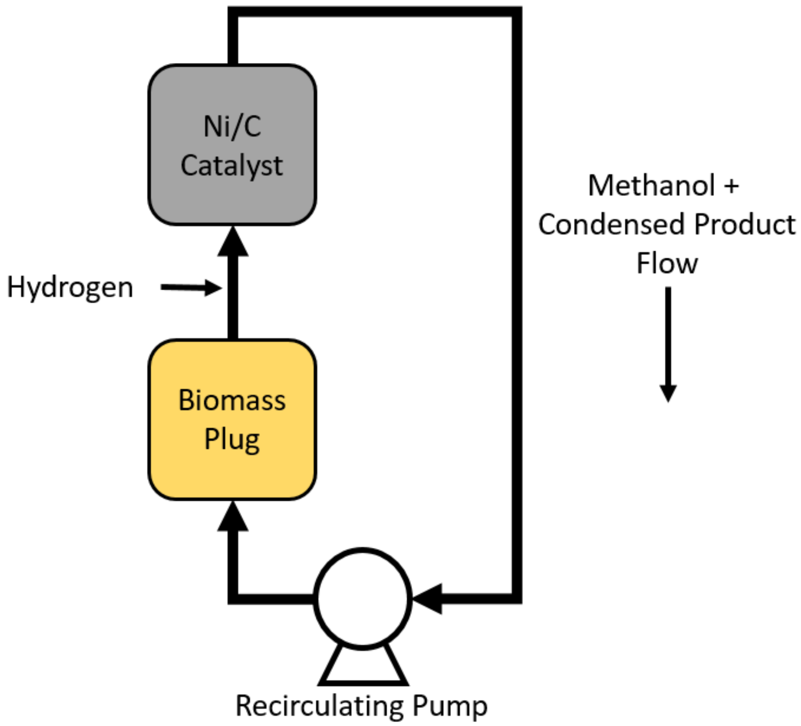

Figure S2. Pump-around reactor design

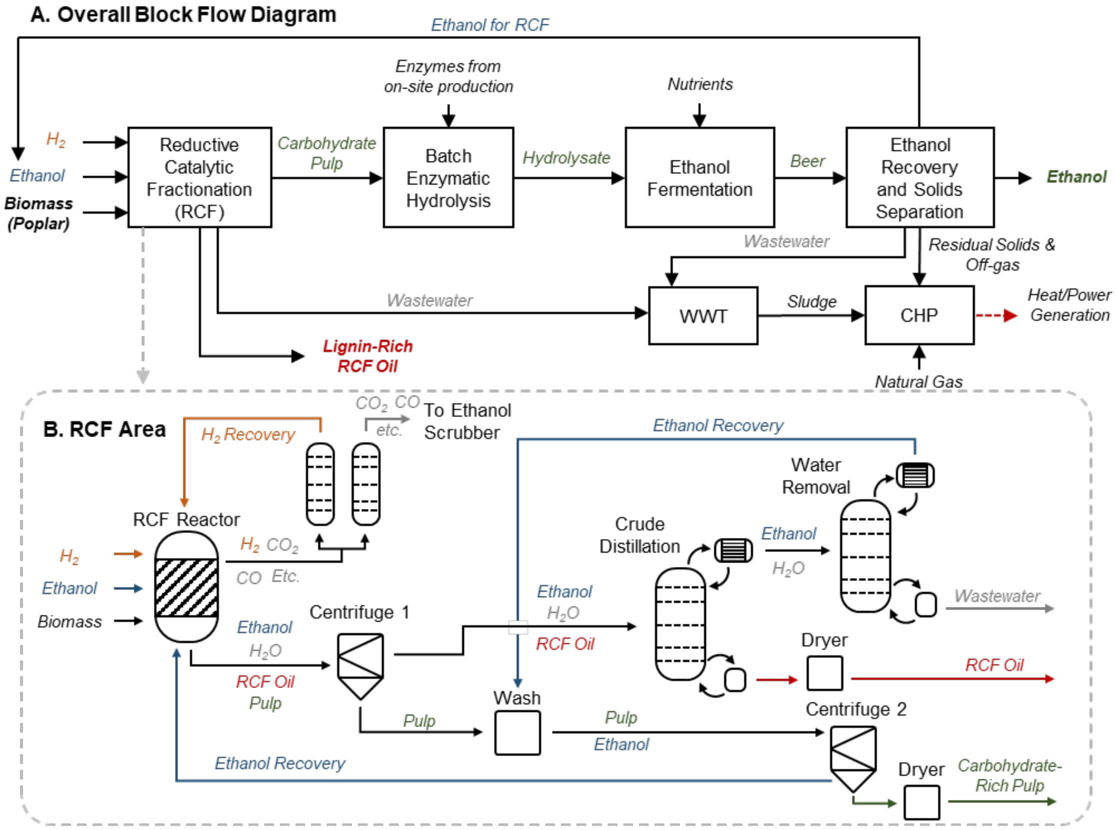

Figure S3. Process Flow Diagram for Ethanol Case

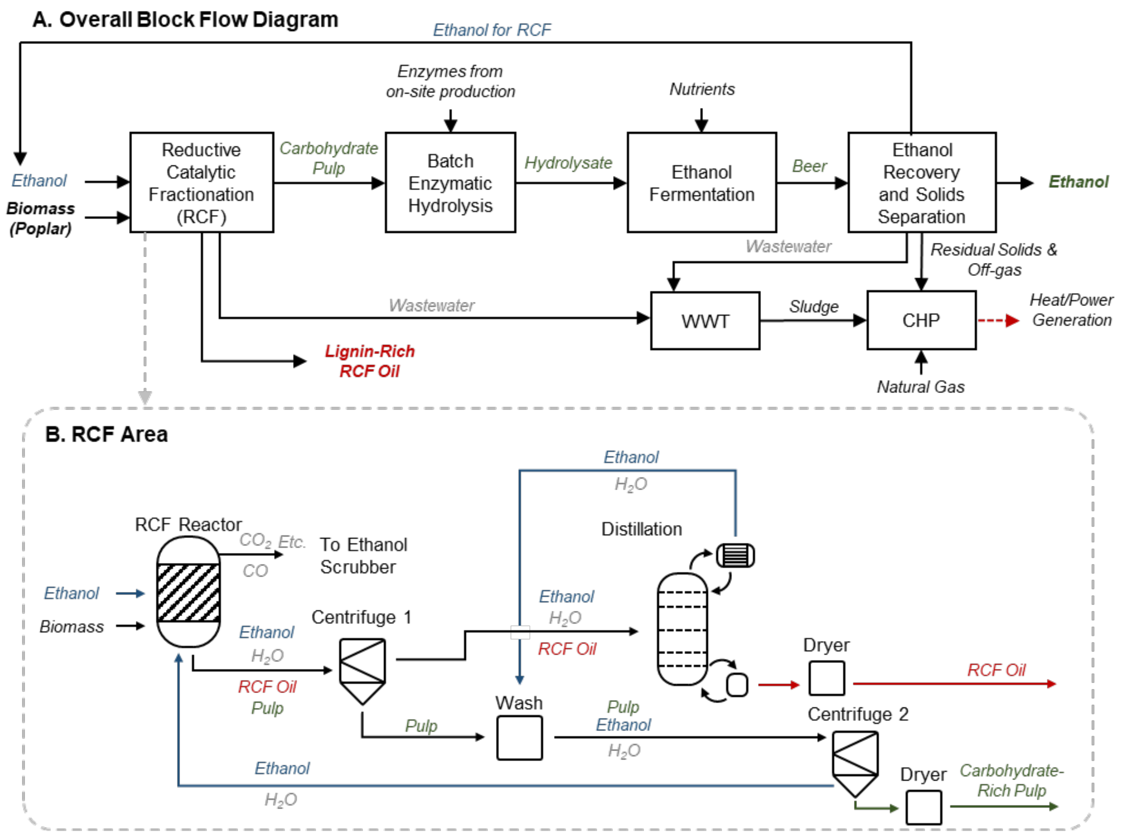

Figure S4. Process Flow Diagram for Hydrogen-Free Case

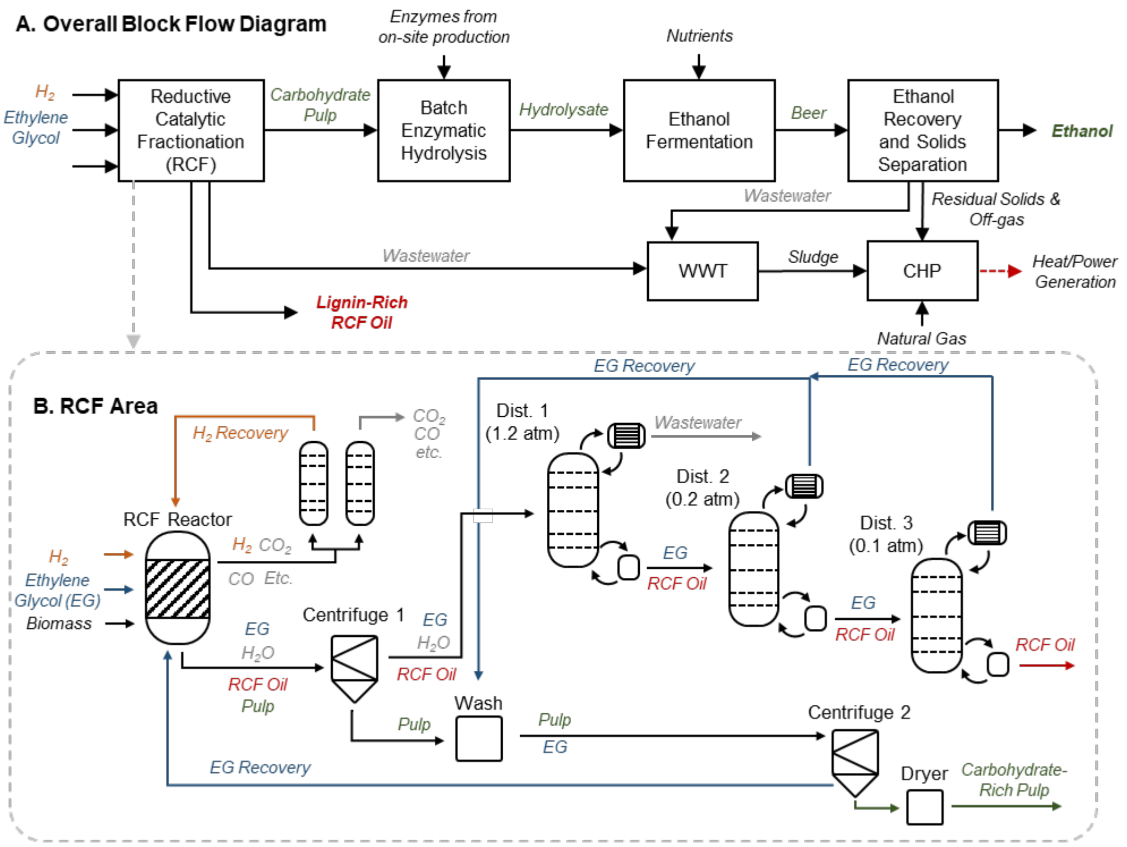

Figure S5. Process Flow Diagram for Ethylene-Glycol Case

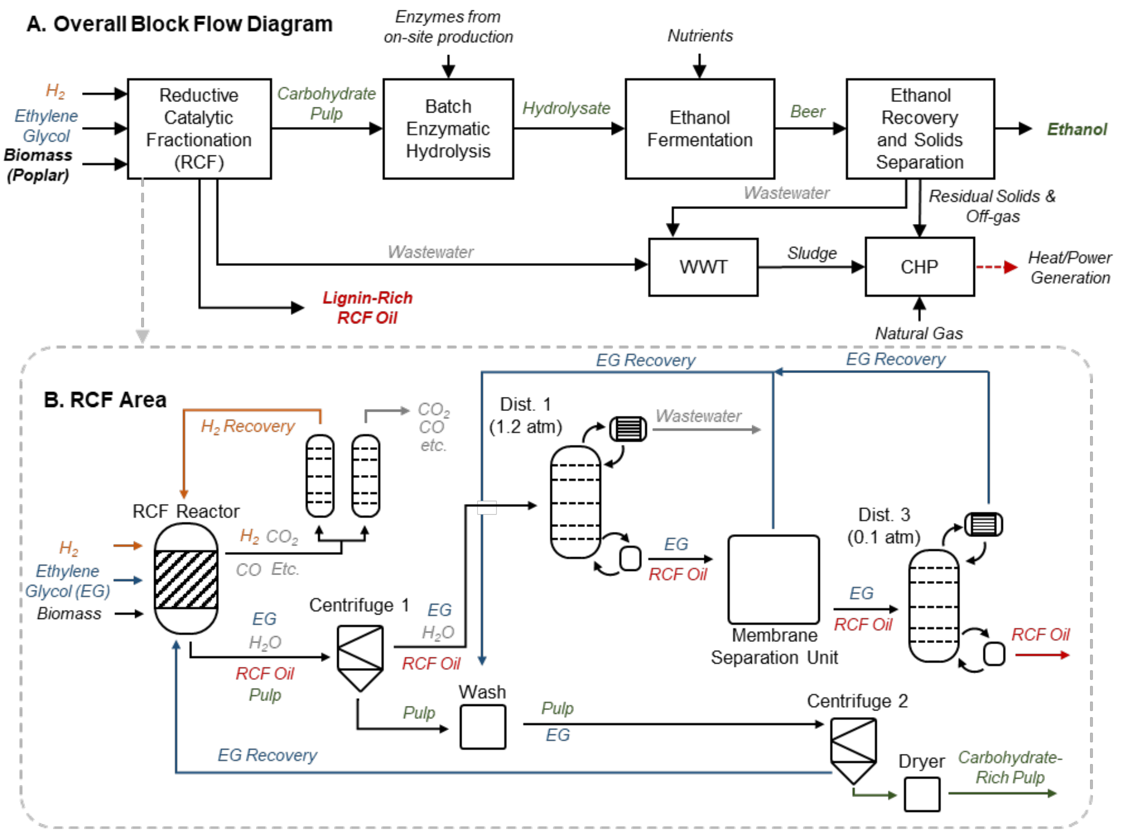

Figure S6. Process Flow Diagram for EG-Membrane Case

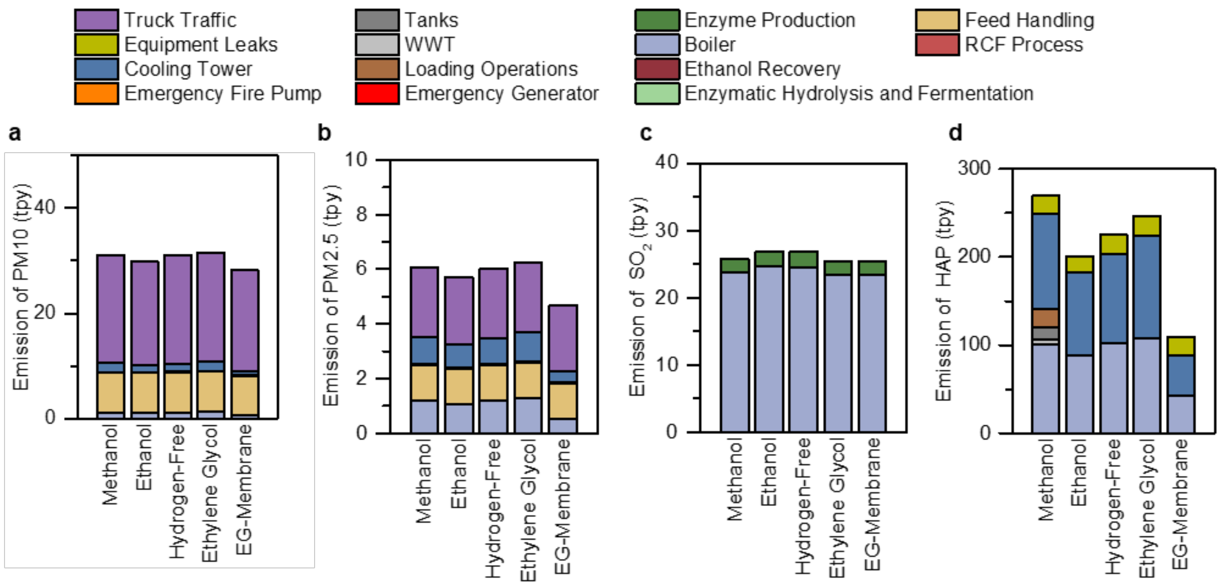

Figure S7. Emissions of (a) PM10, (b) PM2.5, (c) SOx and (d) HAP for each process configuration. See **Tables S23-S32** for tabulated data.

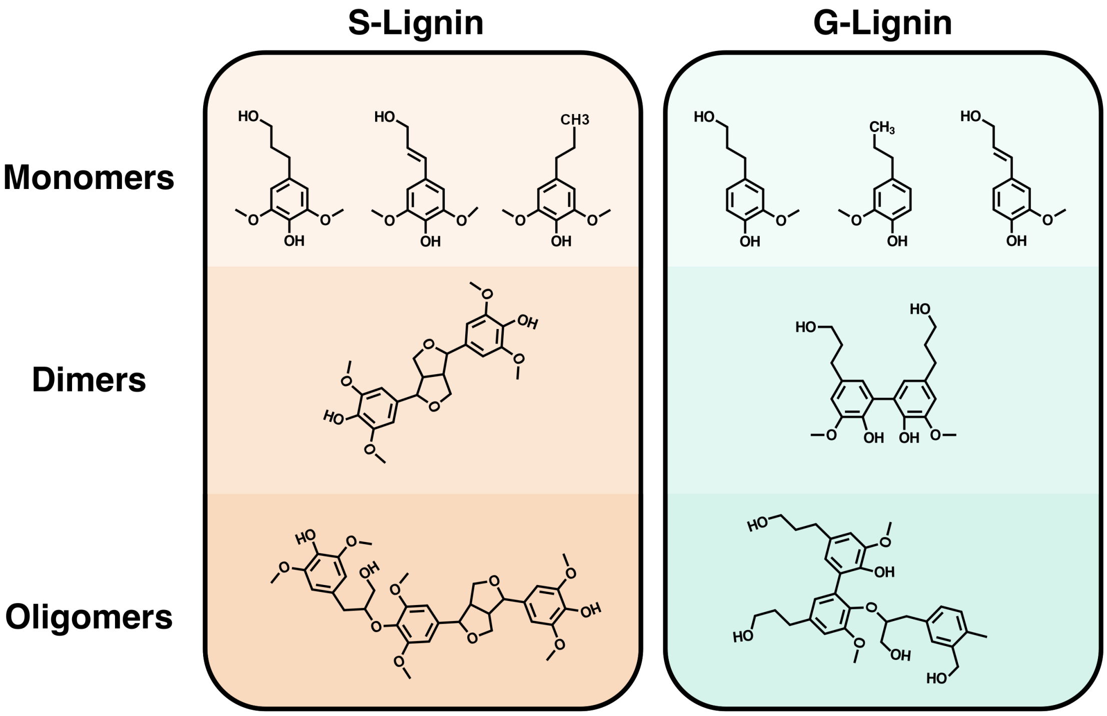

Figure S8. Lignin compounds used in Aspen Plus simulation of the RCF process.

References

1. S. Van den Bosch, W. Schutyser, R. Vanholme, T. Driessen, S. F. Koelewijn, T. Renders, B. De Meester, W. J. J. Huijgen, W. Dehaen, C. M. Courtin, B. Lagrain, W. Boerjan and B. F. Sels, *Energy & Environmental Science*, 2015, **8**, 1748-1763.
2. E. M. Anderson, M. L. Stone, R. Katahira, M. Reed, W. Muchero, K. J. Ramirez, G. T. Beckham and Y. Román-Leshkov, *Nature Communications*, 2019**, 10**, 2033
3. E. M. Anderson, R. Katahira, M. Reed, M. G. Resch, E. M. Karp, G. T. Beckham and Y. Román-Leshkov, *ACS Sustainable Chemistry & Engineering*, 2016, **4**, 6940-6950.
4. R. M. Happs, A. W. Bartling, C. Doeppke, A. E. Harman-Ware, R. Clark, E. G. Webb, M. J. Biddy, J.-G. Chen, G. A. Tuskan, M. F. Davis, W. Muchero and B. H. Davison, *Biofuels, Bioproducts and Biorefining*, 2021, **15**, 176-188.
5. S. Van den Bosch, W. Schutyser, S. F. Koelewijn, T. Renders, C. M. Courtin and B. F. Sels, *Chemical Communications*, 2015, **51**, 13158-13161
6. F. Baddour, K. Van Allsburg, N. Wunder, J. Yarbrough, M. Jankousky, K. Gruchalla, K. Potter, J. Schaidle, E. Tan and M. Talmadge, *CatCost™(Catalyst Cost Estimation Tool)*, National Renewable Energy Lab.(NREL), Golden, CO (United States); Pacific …, 2019.
7. E. M. Anderson, M. L. Stone, R. Katahira, M. Reed, G. T. Beckham and Y. Román-Leshkov, *Joule*, 2017, **1**, 613-622.
8. T. Renders, S. Van den Bosch, T. Vangeel, T. Ennaert, S.-F. Koelewijn, G. Van den Bossche, C. M. Courtin, W. Schutyser and B. F. Sels, *ACS Sustainable Chemistry & Engineering*, 2016, **4**, 6894-6904.
9. M. J. Biddy, C. Scarlata and C. Kinchin, *Chemicals from biomass: a market assessment of bioproducts with near-term potential*, National Renewable Energy Lab.(NREL), Golden, CO (United States), 2016.
